# Single-cell and population level viral infection dynamics revealed by phageFISH, a method to visualize intracellular and free viruses

**DOI:** 10.1111/1462-2920.12100

**Published:** 2013-03-14

**Authors:** Elke Allers, Cristina Moraru, Melissa B Duhaime, Erica Beneze, Natalie Solonenko, Jimena Barrero-Canosa, Rudolf Amann, Matthew B Sullivan

**Affiliations:** 1 Department of Ecology and Evolutionary Biology, University of ArizonaLife Sciences South, 1007 East Lowell Street, Tucson, AZ, 85721, USA; 2 Department of Molecular Ecology, Max Planck Institute for Marine MicrobiologyCelsiusstr. 1, 28359, Bremen, Germany; † Centro de Astrobiología (CSIC/INTA), Instituto Nacional de Técnica AeroespacialCtra. de Ajalvir, km 4, Torrejón de Ardoz, 28850, Madrid, Spain; ‡ University of Michigan, Department of Ecology and Evolutionary Biology, C.C. Little, North UniversityAnn Arbor, MI, 48109, USA; § Authors contributed equally to the manuscript.

## Abstract

Microbes drive the biogeochemical cycles that fuel planet Earth, and their viruses (phages) alter microbial population structure, genome repertoire, and metabolic capacity. However, our ability to understand and quantify phage–host interactions is technique-limited. Here, we introduce phageFISH – a markedly improved geneFISH protocol that increases gene detection efficiency from 40% to > 92% and is optimized for detection and visualization of intra- and extracellular phage DNA. The application of phageFISH to characterize infection dynamics in a marine podovirus–gammaproteobacterial host model system corroborated classical metrics (qPCR, plaque assay, FVIC, DAPI) and outperformed most of them to reveal new biology. PhageFISH detected both replicating and encapsidated (intracellular and extracellular) phage DNA, while simultaneously identifying and quantifying host cells during all stages of infection. Additionally, phageFISH allowed per-cell relative measurements of phage DNA, enabling single-cell documentation of infection status (e.g. early vs late stage infections). Further, it discriminated between two waves of infection, which no other measurement could due to population-averaged signals. Together, these findings richly characterize the infection dynamics of a novel model phage–host system, and debut phageFISH as a much-needed tool for studying phage–host interactions in the laboratory, with great promise for environmental surveys and lineage-specific population ecology of free phages.

## Introduction

Microbes drive the biogeochemical cycles that fuel our planet (Falkowski *et al*., 2008), and their viruses (phages) impact microbes through mortality, horizontal gene transfer and direct manipulation of core metabolisms (Fuhrman, 1999; Breitbart *et al*., 2007; Suttle, 2007). In the oceans this is particularly well studied, but vast knowledge gaps remain. For example, 10–66% of surface water microbes are lysed daily (Fuhrman and Noble, 1995; Steward *et al*., 1996; Suttle, 2007), and we are only just learning that at least as many microbes are infected by integrated ‘molecular time bombs’ (Paul, 2008; phage genomes integrated into the host microbial genome – i.e. a prophage in a lysogen) that eventually lyse the cells as well (Weinbauer, 2004). Specifically, we have learned that cyanobacterial viruses directly impact global carbon cycling. They contain core host photosynthesis genes (*psbA*, *psbD; Mann et al*., 2003; Millard *et al*., 2004) that undergo dynamic phage–host transfer (Lindell *et al*., 2004; Sullivan *et al*., 2006), are expressed during infection (Lindell *et al*., 2005; Clokie *et al*., 2006) for modelled fitness gain (Bragg and Chisholm, 2008; Hellweger, 2009), and are widespread through the surface oceans (of the *psbA* genes in microbial metagenomes with identifiable organismal origins, 60% are encoded by phages; Sharon *et al*., 2007).

Very little interaction data describing environmental phage–host systems are available to the level of detail of the cyanophages. This results from a lack of model systems in culture: of over two dozen known bacterial phyla, only three – *Cyanobacteria* (e.g. Suttle and Chan, 1993; Waterbury and Valois, 1993; Lu *et al*., 2001; Marston and Sallee, 2003; Sullivan *et al*., 2003), *Proteobacteria* (e.g. Wichels *et al*., 1998; Comeau and Suttle, 2007; Zhang and Jiao, 2009) and *Bacteroidetes* (Holmfeldt *et al*., 2007) – have cultured phage–host systems. None are characterized to the detail of cyanophages. Such detail is critical to predict how viruses impact primary production, the microbial loop and carbon cycling (Fuhrman, 1999; Behrenfeld *et al*., 2006; Suttle, 2007), and ultimately, how ocean microbes will respond to global change (Zhou *et al*., 2012). A major cause for this knowledge gap is that virus community measurements do not link uncultured viruses to their hosts. On scales relevant to the complexity observed in the environment, such black box-level measurements leave fundamental questions unanswered, e.g. *how many microbes are infected at any given time, which virus groups are active, and who infects whom*?

Recent studies have advanced our ability to observe such phage–host interactions at the single-cell level. First, microfluidic digital PCR applied to microbes in the termite gut identified specific phage–host associations using individually loaded microbial cells in PCRs targeting both a phage and bacterial gene (Tadmor *et al*., 2011). This represents the best method currently available for experimentally linking a host and its phage without culturing, albeit only by gene–gene colocalization. A second approach applied a method for microbial single gene detection, cycling primed *in situ* amplification – fluorescence *in situ* hybridization, in which target sequences are enzymatically amplified inside the cell and further detected by fluorochrome-labelled probes, to visualize viral DNA transferred from *Escherichia coli* to freshwater bacteria (Kenzaka *et al*., 2010). While significant advances, the former gives neither indication of host cellular morphology nor frequency of infected cells, while for the latter simultaneous identification of the host cells has yet to be shown and visualization of the copy number of phage genomic DNA per cell is not possible. Finally, an elegant microscopy-based study at single-cell (*E. coli*) and single-virus (lambda phage) resolution via fluorescent lambda phage constructs and transcriptional reporters advanced our knowledge of the poorly understood decision between lysis and lysogeny (Zeng *et al*., 2010). However, in most cases, these methods cannot be transferred to exotic, genetically inaccessible environmental systems where our lack of knowledge abounds.

Here we introduce a new method, phageFISH, which is a variant of geneFISH (Moraru *et al*., 2010) optimized for targeting phage. Briefly, geneFISH consists of a gene detection and a rRNA detection step. The gene detection step uses double-stranded (ds) DNA probes labelled with multiple digoxigenin (DIG) molecules, which specifically hybridize to target genes. Further, DIG is recognized by horseradish peroxidase (HRP)-conjugated antibodies. Next, the HRP catalyses the deposition of many fluorescently labelled tyramides in a subsequent catalysed reported deposition (CARD) step. The rRNA detection is achieved by using HRP-labelled oligonucleotide probes to hybridize cellular rRNA, followed by a CARD reaction (CARD-FISH; Pernthaler *et al*., 2002). PhageFISH further develops geneFISH towards (i) detecting phage genes in free virus particles, and (ii) achieving near 100% gene detection efficiency and enabling reliable co-visualization of a phage gene and host rRNA inside infected cells. In this study we apply phageFISH to a one-step growth experiment (Ellis and Delbrueck, 1939) to document phage–host infection dynamics of a novel environmental model system (phage PSA-HP1 and its *Pseudoalteromonas* host) observed in coastal marine environments (Holmström and Kjelleberg, 1999; Wichels *et al*., 2002). A classical one-step growth experiment aims to discretely capture the first cycle of virus production upon introduction of a virus to its host, thus describing the latent period of intracellular phage growth and the average burst size – number of phage produced per host cell (Adams, 1959). To samples of such an experiment, we apply phageFISH to show how its results compare with classical metrics.

## Results

### Optimizing geneFISH to develop phageFISH

While geneFISH has linked cell identity with gene presence in diverse environmental microbes, in samples ranging in complexity from enrichments (Lenk *et al*., 2012), to marine bacterial–eukaryotic symbiotic systems (Petersen *et al*., 2011; Bernhard *et al*., 2012), to upwelling seawater samples (Moraru *et al*., 2010), its application to phage–host systems was limited by a detection efficiency – defined here as the fraction of cells with a positive signal indicating gene presence – of ≤ 40% (Moraru *et al*., 2010). To this end, we increased detection efficiency by increasing the number of polynucleotide probe targets – instead of one 350 bp gene region, up to twelve 300 bp regions of the same gene were targeted [*Supporting information* (*SI*), Table S1]. GeneFISH was performed on *E. coli* cells in which a phage PSA-HP1 gene of unknown function was cloned (genome position 8564–13 387, Fig. S1A; termed here *unk*). The detection efficiency in low target number clones (3–8 copies per cell) ranged from 70 ± 0.2% for one probe to as much as 98 ± 0.1% for 12 probes (Fig. S1B). In high target number clones (up to 200 copies per cell), 92 ± 1.4% of the targets were detected using only one probe and nearly 100% with ≥3 probes (Fig. S1C). We next varied the dextran sulfate (DS) concentrations in the catalysed reporter deposition (CARD) step (see *SI Text*) to optimize the relationship between the gene signal size and target number. We found that 20% DS concentration gave the sharpest gene signal (Fig. S1D), highest detection efficiency and acceptable noise level (Fig. S1E). Moreover, it gave a dot-like signal for single copy targets and a larger signal (cell spread) for high copy targets, even when hybridized with a large number of probes (Fig. S1F). For application to one-step growth experiments (below), the optimized protocol (phageFISH) used a combination of six probes and 20% DS.

### Phage–host dynamics during a one-step growth experiment

To evaluate the performance of phageFISH against standard metrics, we characterized the dynamics of podovirus PSA-HP1 infecting *Pseudoalteromonas* sp. strain H100 in a one-step growth experiment. It is not yet known conclusively whether PSA-HP1 is capable of an integrated mode; however, there are no genes indicative of a temperate lifestyle in the sequenced genome (M.B. Duhaime, unpubl. data).

**Figure 1:**
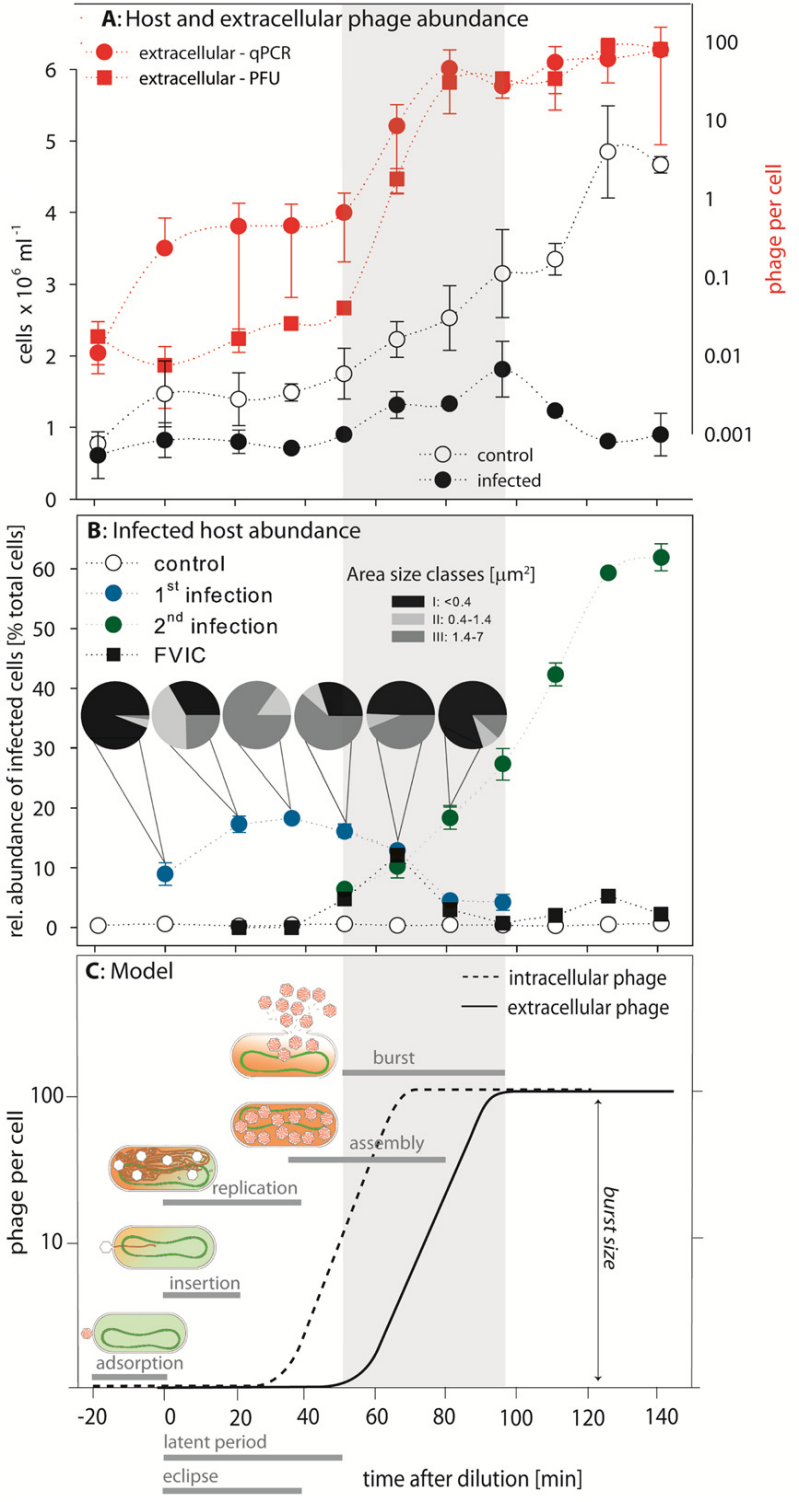
One-step growth infection dynamics of a marine virus–host system.A. Host abundance as total cell counts and extracellular virus abundance as virus abundance per cell by gene presence (qPCR) and plaque assays (PFU).B. Relative abundance of phage containing cells as FVIC by TEM count (black squares) and phageFISH count (coloured circles); pie charts indicate area size distribution among phage signals as determined by phageFISH.C. One-step growth model for phage PSA-HP1 and its host H100.Error bars indicate standard deviation except for FVIC data in which error bars indicate 95% confidence intervals. All data are based on measurements from two biological replicates. All data at T-19 are corrected by a 1/100 factor to be comparable to values measured after dilution of cultures.

The one-step growth experiment proceeded as follows: after physiological acclimation (*SI Text* and Fig. S2), *Pseudoalteromonas* cells were incubated 19 min with half as many phages (Multiplicity Of Infection, MOI = 0.5), diluted 100-fold to minimize further phage adsorption, and sampled immediately after dilution (T0; time points are denoted with a ‘T’ followed by the number of minutes post-dilution). Infected host cultures increased in cell abundances until T96, but their growth was compromised as compared with control cultures (Fig. 1A). Free phage numbers rose from T51 to T81, as detected by qPCR-based assays of extracellular phage genomic DNA (qEXT) and plaque-forming unit (PFU) assays of infective phage particles (Fig. 1A, controls see Fig. S3). Intracellular, mature phage particles were first detected at T36 using transmission electron microscopy (TEM), which marks the end of phage DNA replication at T36. The first visibly infected cells (defined as ≥5 phage particles per cell; Brum *et al*., 2005) were observed by TEM at T51, and the frequency of visibly infected cells (FVIC, see *Experimental procedures*) reached a maximum at T66, when 12.1 ± 0.02% (confidence interval, c. i.) of cells were infected, after which FVIC decreased to 3.0 ± 0.01% (c. i.) and remained low for the remainder of time points (Fig. 1B).

### Detailed phage–host dynamics observed with phageFISH

**Figure 2:**
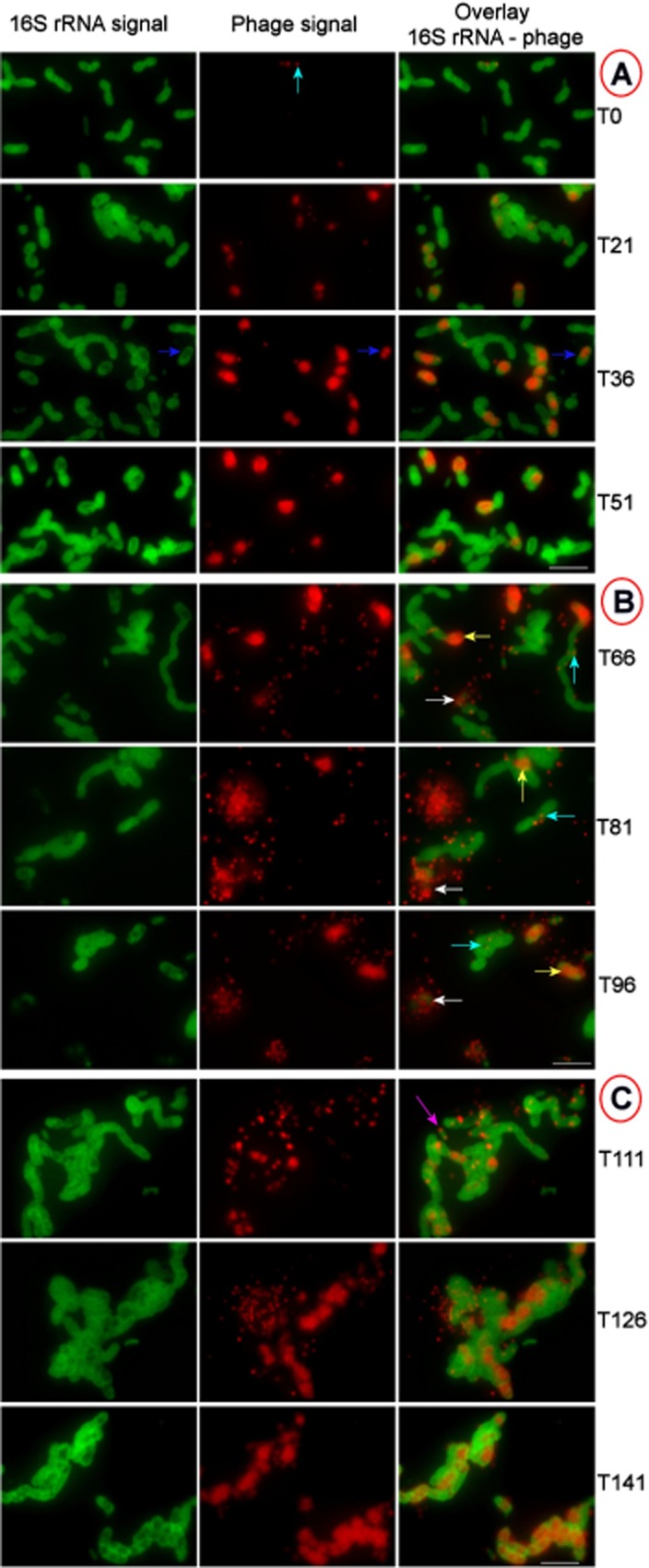
Progression of infection over time: epifluorescence micrographs of virus-infected and uninfected host cells after phageFISH.A: T0–T51, B: T66–T96, C: T111–T46.Left: host only. Centre: virus only. Right column: overlay of host cells in green (Alexa_488_) and virus in red (Alexa_594_).Colour designation for arrows: white = cell lysis and release of free phages, cyan = new infection, blue = rRNA and phage localization, yellow = advanced infection, violet = infection by two phage. The scale bar indicates 5 μm.

PhageFISH provided two metrics in this one-step growth experiment: (i) a quantitative metric – the fraction of infected cells, and (ii) a semi-quantitative metric – the relative extent of per-cell phage infection, measured as the area of the phage signal. The latter ranged from a fraction to the entire cell (see Fig. 1B, pie diagrams, and Fig. 2) and was grouped into three distinct size classes (for details, see Fig. S4): (I) < 0.4 μm^2^, which was the majority of the infections at T0 and most likely represents new infections, (II) 0.4–1.4 μm^2^, which most likely represents the viral DNA replication stage, and (III) 1.4–7 μm^2^, which was the majority of the infections at T36, and most likely represents advanced infections (late replication and assembly).

The combination of the two metrics allowed us to discriminate between two waves of infection and to describe the infection stages within them (for details on how the two infection waves were segregated, see Fig. S4). The first wave had phage signals that grew from size class I at T0 to size class III (see Fig. 1B – blue circles and, Fig. 2A and B) up to and including cell lysis at T51 (but as late as T96, see below). The second wave signals appeared initially at T51 (as size class I) and grew (both in numbers and in signal area) until T146 (see Fig. 1B – green circles and Fig. 2B and C). Further, within each wave of infection we were able to document details of their relative infection stages. For example, first wave infections plateaued at T21 with 17.3 ± 1.4% (std. dev.) of the cells infected, but reached the largest phage signals (size class III) at T36 (pie charts in Figs 1B and 2A). This indicates that few or no new infections were initiated post-T21, but that old infections were progressing via intracellular genome replication until T36. Starting with T51, the decline of the first wave and the onset of the second wave of infection are indicated by (i) the appearance of free phage particles due to cell lysis events (Fig. 2B, described further below), *(*ii*)* the decrease in the fraction of cells with advanced infections (size classes II and III), which was minimal at T81 (4.3 ± 1.3% st. dev.) and (iii) the shift back toward smaller signal size classes (Figs 1B and 2B), indicative of new infections. The per-cell phage signal area allowed simultaneous tracking of these two waves of infection as they overlapped from T51 until T96, by which time even the latest of the first wave-infected cells had lysed. Notably, through the whole experiment, the phage signal showed distinct sub-cellular localization in the middle of the cell, while the rRNA signal localized at the periphery (Fig. 2), a feature further supported by TEM images of infected cells (Fig. S5).

**Figure 3:**
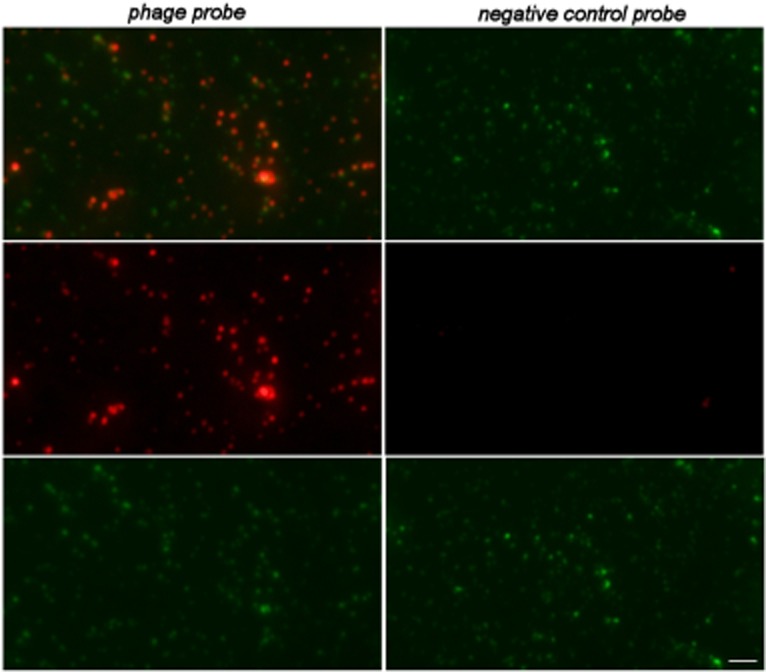
Epifluorescence micrograph of free virus after phageFISH (red, Alexa_594_) and SYBR staining (green).On the left: probe targeting a viral gene, on the right: negative control gene probe.Top: overlay of SYBR Green and phageFISH, centre: phageFISH only, bottom: SYBR Green only. Scale bar indicates 2 μm.

In addition to these intracellular metrics, phageFISH also detected phage DNA extracellularly, in free phage particles. Simultaneous with the onset of the second wave of infection, we observed phageFISH-stained extracellular particles associated with lysed cells (Fig. 2B and C), suggesting that phageFISH can label target DNA encapsidated in free phage particles. While neither our original intention nor expectation, two follow-up experiments confirmed that phageFISH could indeed target free phage particles. First, a negative control gene probe (Moraru *et al*., 2010) applied to infected cells showed only ∼ 2% of the cells having false positive signals of the lowest size class, while no cell lysis-like events were visible (Fig. S6), indicating that the phageFISH-stained extracellular particles represented specific hybridizations of the phage probes to DNA. Second, phageFISH on a 0.2 μm-filtered lysate showed clear association between the phageFISH and phage DNA stain (SYBR Green) signals, while no hybridization signals were observed for the negative control gene probe (Fig. 3; for further details on the negative control gene probe, please see *SI Text*, *phageFISH* section).

### Towards a model of one-step growth in this new phage–host system

The dynamics of phage PSA-HP1 infecting *Pseudoalteromonas* sp., as modelled based on phageFISH data, matched the dynamics per combined PFU, qPCR and FVIC data, as summarized in Fig. 1C. Notably, lacking the power to quantitatively discern discrete intracellular phage signals, phageFISH does not yet allow for estimates of burst size; those estimates were derived from the qPCR and PFU data (see below). After 19 min of adsorption (T0) and within 21 min post-dilution (T21), the phage successfully inserted its DNA into ∼ 17% of the host cells (phageFISH – Fig. 1B, blue circles). Simultaneously, starting in the first phage-adsorbed cells, new phage DNA was replicated intracellularly until T36 (FVIC – Fig. 1B, or phageFISH – Fig. 1B, phage signal per cell area), with assembly of new progeny finished by T66 (FVIC). The first cells started lysing by T51, with the main lysis event occurring between T66 and T81 (qEXT and PFU – Fig. 1A, or phageFISH – Figs 1B and 2B). Together this suggests a latent period of 51–66 min for phage PSA-HP1 and an overall phage generation time (adsorption plus latent period) of 70–85 min. In addition, as cells lyse, the per-cell phage signal area measurements resulted in immediate detection and discrimination between the first and second waves of infection (Figs 1B and 2B). FVIC was not used here to determine burst size, as the cells underwent histological embedding and thin-sectioning for imaging. Instead, the burst size was estimated by PFU to be 100 ± 0.3 phages per cell, and by qEXT to be 98 ± 0.3 phages per cell.

## Discussion

### Methods optimizations

PhageFISH retains the ability to identify host cells by rRNA CARD-FISH, while improving upon current methods of single gene detection by (i) increasing gene detection efficiency, (ii) establishing target signal areas, which enables simultaneous visualization and relative quantification of the intracellular phage DNA signal, and (iii) detecting phage DNA in free phage particles.

Previous FISH-based methods for gene detection have low detection efficiency, ranging from 15% (Hoshino and Schramm, 2010; Kawakami *et al*., 2010) to 40% (Moraru *et al*., 2010). Recently, Kawakami and colleagues (2012) have improved detection efficiency to 98% by both increasing the length of the probe (up to 820 bp) and adding a second CARD step. However, this method has yet to be combined successfully with cell identification by rRNA CARD-FISH, most likely because the application of two CARD steps for gene detection leaves little space in the cell for tyramides used in a third CARD step for rRNA. Moreover, the method gives a whole-cell gene signal, and thus lacks the resolution to distinguish discrete stages in the phage–host infection cycle that often occur at sub-cellular level (e.g. integrated single-copy phage genome versus multicopy phage genome during active lytic replication). The benefit of a focused sub-cellular signal is that it can reflect, e.g. phage genome copy number per cell as it increases during infection.

In comparison, our strategy consisted of a gradual increase in target length by using multiple short polynucleotide probes, instead of one long probe. This allowed an increase in the detection efficiency while minimizing probe penetration-related problems (the longer the probe, the more difficult to penetrate the cell) and gene signal diffusion, which results from the second CARD protocol. As expected, the detection efficiency in the low copy clones (3–8 copies per cell) increased sharply from ∼ 70% for one probe to > 90% for four probes, and then slowly towards ∼ 98% with each probe added until 12. For clones with 1–2 copies per cell and one probe, the geneFISH protocol had ∼ 40% efficiency (Moraru *et al*., 2010). Theoretical calculations (see *SI Text*) indicate that at least five probes are necessary for > 90% detection for 1–2 copies per cell, and at least 12 probes for one copy per cell. Overall, this indicates that the number of probes needed increases with a decreasing number of targets per cell. When the phage genome is single copy (immediately after infection), at least 12 probes are needed. Once the phage genome begins to replicate, fewer and fewer probes are needed for high detection efficiency, e.g. four probes for 3–8 genome copies per cell and one probe for > 50 copies per cell. This range is biologically relevant for environmental observation, as, in marine phage–host systems, the number of phages per cell generally ranges from 1 to 600 for pure cultures and from 20 to 50 in the environment (Borsheim, 1993; Weinbauer and Peduzzi, 1994; Wilhelm *et al*., 1998).

Our efforts to work with a focused phageFISH signal at varying target concentrations (Fig. S1D and F) represent a first step towards discrimination between different phage infection stages through relative quantification of the per-cell phage signal area (details and limitations below).

For the purpose of this experiment, we have used six probes, which result in a detection efficiency of > 92% for cells with at least 3–8 phages per cell (during replication). As such, we expect an underestimation of the number of infected cells for cells with only one phage, and a good estimation as soon as the phage replication starts.

### PhageFISH provides novel insights into phage–host dynamics

PhageFISH describes the phage infection in a higher degree of detail than any of the other methods used here for comparison. First, phageFISH allowed us to build a model of the previously uncharacterized infection dynamics between phage PSA-HP1 and *Pseudoalteromonas* sp. strain H100 (Fig. 1C), with the exception of the burst size, as explained above. Our observed phageFISH metrics (Fig. 1B) were corroborated by classical measurements, as the temporal dynamics of the phageFISH signal is consistent with qPCR- and PFU-based measurements of viral abundance (compare Fig. 1A and B) and FVIC counts (Fig. 1B). The latent period (51–66 min; Fig. 1) and phage generation time (70–85 min; Fig. 1) for PSA-HP1 on *Pseudoalteromonas* H100 are in the range of other marine phage–host systems studied in the lab (e.g. cyanopodovirus Syn5, Raytcheva *et al*., 2011, and phages infecting various marine heterotrophs, Jiang *et al*., 1998), while others, such as marine T7-like cyanopodovirus infecting *Prochlorochoccus* MED4, are known to require 8 h for phage production (Lindell *et al*., 2007). This variation in timing of lysis is thought to be a consequence of a trade-off between host quantity and quality (Wang *et al*., 1996), and thus is likely to be variable among environmental populations as well.

Second, phageFISH outperforms the other methods employed to monitor the phage infection dynamics. For example, while PFU and extracellular phage gene qPCR describe the infection stages moderately well (see Fig. 1A), they lack the ability to measure the fraction of infected host cells and fail to discriminate between the two waves of infection. Additionally, the more traditional FVIC metric successfully reports the fraction of visibly infected host cells, but is limited in that it can detect only late stage infection (mature viral particles), misses the second wave of infection, and, while only a problem for application to mixed communities, does not provide lineage-specific information. PhageFISH succeeds on all these fronts. Moreover, even where measurements can be similarly made, phageFISH is likely to be more sensitive than, e.g. FVIC due to its ability to detect phage replication through the entirety of the lytic infection. Specifically, we found that FVIC underestimated the maximum fraction of infected cells (at T66: 12.1 ± 0.02% c. i.) relative to phageFISH (at T21: 17.3 ± 1.4% std. dev.). Presumably, this is due to the fact that phageFISH detects intracellular viral DNA (both free and encapsidated) present through the infection, while FVIC is able to detect *assembled* virus particles present in the cells only in late-stage infection. In our experiment, by the time FVIC peaks at T66, part of the first-wave-infected cells have already lysed and released new phage (Fig. 1A), resulting in FVIC under-documenting the total fraction of infected cells from the first wave, as would be expected in a semi-synchronized population (Proctor and Fuhrman, 1990; Proctor *et al*., 1993). This is supported also by phageFISH, which, at T66, shows that 12.9 ± 0.6% std. dev. infected cells for the first wave. Additionally, the centrifugation step of the protocol for TEM samples could cause disruption of infected cells and thus lead to lower counts, as was contemplated by Weinbauer and Höfle (1998).

In addition to intracellular, single-cell detection capabilities, phageFISH also enables detection of free phages. This was most likely made possible due to various steps in the phageFISH protocol capable of denaturing proteins and nucleic acids, e.g. acid, SDS, and formamide and high temperatures (see *SI Text*, *phageFISH* section), thus rendering the virus particles accessible to probes. In our experiment, the detection of free phages was particularly valuable for careful documentation of the timing of cell lysis. For example, we observed free, presumably new, phages near cells that appeared to be lysed – i.e. cells with weaker 16S rRNA signal and/or broken cells (Fig. 2B). Almost simultaneously, the bacterial cells started to form aggregates, likely due to the secretion of extracellular polymeric substances. We suspect that these sticky extracellular secretions and cell debris of lysed cells caught a fraction of the free phage and prevented them from passing through the 0.2 μm pore-sized filters during sampling in late-stage infections (e.g. T51), while in early-stage infections (e.g. T0), neither cell debris, nor extracellular secretions, nor free phages were observed.

### Current phageFISH applications

PhageFISH holds great promise for advancing viral ecology. First, phageFISH used in traditional model system experiments can advance the field one phage–host system at a time, as accomplished here. Beyond lytic infections, phageFISH offers a particularly valuable tool for studying temperate phage infections, which are now thought to be prevalent in marine systems as referenced by Paul (2008). Even while relatively quantitative, phageFISH allows a researcher to monitor when a temperate phage has switched from a single-copy, silent prophage state to a replicating lytic state.

Second, with improved sensitivity over geneFISH, we are confident phageFISH can be applied successfully to environmental samples, in a similar manner as geneFISH (Moraru *et al*., 2010; Petersen *et al*., 2011; Bernhard *et al*., 2012). Here, simultaneous visualization of phage genes and host rRNA offers a culture-independent means to answer, for targeted populations: *how many microbes are infected at any given time, which phage groups are active, and whom do they infect?* Such lineage-specific frequency-of-visibly-infected-cell measurements are pivotal steps towards linking virus and host community metrics (e.g. counts, production assays) with community sequence data, which have furthered our understanding of the identities and dynamics of key members of both communities already. Third, as FISH transformed environmental microbiology (*sensu* Amann *et al*., 1995), phageFISH enables sequence-based population ecology of free phage particles in the environment, as no current method allows.

While the application of phageFISH to the environment requires prior knowledge of phage and host gene sequence variation for probe design, such data are becoming routine as metagenomic sequencing improves and scales up. Even probe design for the large sequence diversity of environmental phages should prove surmountable by a combination of strategic target group choices and experience gleaned from polynucleotide probes targeting functional genes using geneFISH (Moraru *et al*., 2010 2011). Unlike cellular organisms, which share universal marker genes and several core genes, the most widely shared gene among viruses is found in only 37% of sequenced viral genomes (Kristensen *et al*., 2012). Investigation of orthologous genes in viruses has found that 21 of 57 tested viral taxa are represented by at least one signature gene (Kristensen *et al*., 2012) that (i) is present in all members of the taxon (a measure of signature gene ‘sensitivity’), (ii) does not exist outside the taxon (signature gene ‘specificity’), (iii) is virus-specific, and (iv) is single-copy in the viral genomes. These over 100 taxon-specific signature genes (Kristensen *et al*., 2012), plus alternatively determined core genes of T4-like myoviruses (Sullivan *et al*., 2010) and T7-like podoviruses (Labrie *et al*., 2013), can be further evaluated for phageFISH polynucleotide probes development (Moraru *et al*., 2011), to determine those which can tolerate up to 10–15% mismatches while still retaining their taxa specificity and sensitivity. Recent work examining T4-like virus genomic diversity suggests this mismatch tolerance to be relevant for both within-population (< 5% gene divergence) and between-population (10–15%) investigation ((L. Deng, J.C. Ignacio-Espinoza, A. Gregory, B.T. Poulos, P. Hugenholtz, M.B. Sullivan, submitted)). However, such family-specific gene marker studies are not without issue (reviewed in Duhaime and Sullivan, 2012). In particular, this approach suffers from limited and taxa-biased virus sequence databases (Kristensen *et al*., 2012), which will affect the power of phageFISH probe recall and precision and will require prudent consideration before application to viral populations in the environment.

### Limitations and opportunities: the phageFISH crystal ball

The ability of phageFISH to quantify relative per-cell phage DNA copy number (e.g. by phage signal area) presents an opportunity for discovery of novel infection and cell biology features and phenomena. Here, it allowed us to document heterogeneity within the infected population and to visualize sub-cellular features of single cells, as we were able to resolve non-overlapping signals for phages and host ribosomes (Figs 2A and S5). This portends the study of cell biology of phage-infected microbes in ways not previously possible.

One current limitation to such absolute quantification is the CARD step (see *SI Text*). The amplified fluorescent CARD signal results from HRP activating labelled tyramides to bind to tyrosine residues in cellular proteins (Bobrow *et al*., 1992), with the number of HRP being related to the number of target-bound probes. Such a signal is likely to remain relatively linearly correlated to target copy number until the cellular environment becomes limited in tyramide binding sites (e.g. tyrosine residues in cellular proteins). Thus, broad size classes (e.g. pie charts in Fig. 1B) estimate phage infection progress at the single-cell level in model systems, but finer-scale infection dynamics and/or more complex community application will require more absolute quantification of this per-cell-area signal. Super-resolution microscopy (Schermelleh *et al*., 2010) might be the answer, as its higher sensitivity and resolution are likely to alleviate the need for CARD amplification altogether. Recently, super-resolution microscopy has allowed the quantification of ribosomes in *E. coli* based on detection of autofluorescent fusion proteins (Bakshi *et al*., 2012). Moreover, the first steps in combining FISH techniques and super-resolution microscopy in microorganisms have already been made and led to improved sub-cellular localization of rRNA, including the detection of sub-cellular areas of high versus low ribosomal content (Moraru and Amann, 2012). A CARD-free phageFISH protocol, combined with sub-cellular localization and quantification by super-resolution microscopy, would refine our understanding of the phage lytic process at stages where the numbers of per-cell phage DNA copies and encapsidated phages are informative and it could even allow differentiation of cells with actively replicating phages (concatenated DNA and unordered phage signal; Fig. 1C ‘replication’) versus cells with encapsidated phage DNA (structured localization of phage signal; Fig. 1C ‘assembly’). Both single-cell genome replication rates and burst sizes could be documented across a heterogeneously impacted host cell population, and, if performed on environmental samples, could be done in a lineage-specific manner. PhageFISH with absolute per-cell phageDNA copy quantification could further transform our understanding of lysogeny and its environmental significance (Weinbauer, 2004), e.g. by targeting well-conserved, known families of temperate phages to provide both quantitative estimates of the fraction of cells infected by single-copy (prophage state) and multiple-copy (lytic infection) phages. Further, such ecological descriptions coupled to phageFISH-enabled laboratory experimentation would undoubtedly advance our mechanistic understanding of a key feature of temperate phages – the factors controlling the lytic/lysogenic switch (Zeng *et al*., 2010; Ptashne, 2011).

### Conclusions

Viruses are fundamental to ecosystem dynamics, yet viral ecology is bottlenecked by insufficiently documented biases in traditional methods and the need for new techniques to study virus–host interactions. On the former, only recently are data available to understand how concentration, purification, and amplification strategies impact viral metagenomes (Duhaime *et al*., 2012; Hurwitz *et al*., 2012). On the latter, new methods – e.g. microfluidic digital PCR (Tadmor *et al*., 2011) and viral-tagging (Deng *et al*., 2012) – are emerging, which may finally experimentally link uncultured viruses to their hosts on scales relevant to examining population dynamics in complex environments. Additionally, viral data emerging from single-cell (e.g. Yoon *et al*., 2011), and now single-virus (Allen *et al*., 2011), sequencing projects provide data on newly described and existing viral types. PhageFISH complements these approaches by simultaneously characterizing phage lytic cycle features at both single-cell and population level resolution, while maintaining knowledge of the virus–host interaction. Together these new tools – by mapping the virus–host interaction landscape – should provide the fundamental data needed to improve our ability to predictively model the dynamics between the two most abundant biological entities on Earth.

## Experimental procedures

### One-step growth experiment

*Pseudoaltermonas* sp. strain H100 (Wichels *et al*., 1998) was grown in 20% nutrient Zobell marine media for three consecutive generations to ensure a consistent growth rate, with a doubling time of approximately 80 min during exponential growth (see *SI Text* and Fig. S2). The last overnight culture was transferred 1:10 to a new 60 ml culture. After 3 h of growth (early exponential phase; Fig. S2), duplicate host cultures were mixed with phage PSA-HP1 at an MOI of 0.5 (phage titres estimated by PFU). Phages were adsorbed to hosts for 19 min, then diluted 1:100 with media to reduce the likelihood of additional adsorption to obtain a near-synchronized infection across the population (see *SI Text*).

### Classical metrics

*Phage numbers* were quantified by SYBR Gold staining (see *SI Text*) prior to infection and by quantitative PCR (qPCR) and PFU during the experiment (see *SI Text*). For qPCR, primers targeted a single copy, non-coding sequence motif from the PSA-HP1 genome. For PFU, plaques were enumerated on a host lawn grown on agar plates (described in *SI Text*). *Burst size*, the number of phage progeny resulting from the infection of one host cell, was estimated by dividing the total burst size averaged over the last five time points of the one-step growth experiment by the number of infective phage. Since the observed phage numbers (Figs 1A and S3) were determined by different methods, i.e. plaque assay (for PFU) and qPCR (for gene copy number), two different approaches were followed to calculate the number of infective phages. The PFU-based number of infective phages was estimated by subtracting the number of extracellular phage present after dilution (PFU data, average of the first three time points) from the number of phage that were added initially as observed by PFU (i.e. burst size via PFU). The qPCR-based number of infective phages was estimated by subtracting the number of extracellular phage present after dilution (qPCR data, average of the first three time points) from the number of phage that were added initially as counted microscopically after SYBR staining (i.e. burst size via qPCR). *Total bacterial cell numbers* were microscopically evaluated after staining the immobilized host cells with 4′,6-diamidino-2-phenylindole (DAPI) (see *SI Text*). For a count of FVIC (Proctor *et al*., 1993), samples were fixed and prepared for TEM as described in *SI Text*. For each time point, the first 800 intact cells from one biological replicate were examined at 32 000–88 000 magnification. If the number of mature viruses in a cell was > 5, it was scored as infected (Brum *et al*., 2005).

### phageFISH

geneFISH optimization and phageFISH on *Pseudoalteromonas* cells and on phage lysates are described in detail in *SI Text*. Briefly, polynucleotide probes for gene detection were synthesized by DIG-dUTP incorporation during PCR. The stringency conditions for hybridization were determined by *in vitro* measurements and calculations (see *SI Text* and Table S3). Then, after sample fixation, immobilization, permeabilization and inactivation of endogenous peroxidases, rRNA was detected by hybridization with a HRP-labelled oligonucleotide probe (EUB338; Amann *et al*., 1990) and a subsequent CARD of Alexa_488_-tyramides (see *SI Text*). For gene detection, first the mRNA was digested and the HRP introduced in the rRNA step inactivated. Then, the *unk* gene was hybridized, followed by binding of DIG by HRP-labelled antibodies and CARD of Alexa_594_-tyramides (see *SI Text*). For phageFISH on phage lysates, the rRNA detection and mRNA digestion steps were omitted and the same *unk* gene was hybridized (see *SI Text*). Samples were counterstained with DAPI or SYBR Green and analysed by epifluorescence microscopy (see *SI Text*). The *unk* gene was chosen as target for phageFISH as it was unique to the phage genome (not in the host genome) and because it was long enough to accommodate 12 probes of 300 bp each, which were needed for the detection efficiency tests.

All detailed protocols, in addition to being available in the SI of this paper, can also be found at http://eebweb.arizona.edu/Faculty/mbsulli/protocols.htm.

## Supporting Information

**SI Text.** **Table S1.** Polynucleotide probes targeting a region spanning a phage gene of unknown function (abbreviated here as unk) in the *Pseudoalteromonas* phage PSA-HP1 genome (genome position 8564-13 387 bp). **Table S2.** Q-PCR primer and amplicon sequences. **Table S3.** Calculated and measured T_m_ for the polynucleotides forming the unk probe mix. **Fig. S1.** geneFISH protocol optimization. A. Genome map of phage PSA-HP1. The six (300 bp each) probe-target regions are indicated in orange. Probes target unknown phage gene, *unk* (grey). B. Variation of the gene detection efficiency with increasing number of polynuclotide probes. *Escherichia coli* low target copy clones (3-8 copies per cell) were hybridized with an increasing number of polynucleotide probes targeting the *unk* gene. As negative control (no *unk* gene), *E. coli* strain B/R cells were used. The detection efficiency is defined as the fraction of all cells showing a gene positive signal. C. Detection of unk gene in high target copy cells using three polynucleotide probes - all the cells have a gene signal, resulting in 100% detection efficiency. Top image - overlay image between 16S rRNA signal and gene signal. Bottom image - gene signal. Scale bar = 5 μm. Exposure time (ms, milliseconds) is described for the gene image. D. Appearance of gene signals for different dextran sulfate concentrations (10%, 20% and 30%). All pictures were taken using the same exposure time. Concentrations of 20% and 30% dextran sulfate resulted in a much sharper signal as compared with 10%. Scale bar = 5 μm. E. Gene detection efficiency for different dextran sulfate concentrations (10%, 20% and 30%). Blue bars = low target gene copy cells, red bars = negative control cells. While the detection efficiency was high for all concentrations, the background level (% of false positives in the negative control) increased with the dextran sulfate concentration. F. Variation of the gene (unk) signal intensity and spread through the cell with variation of the gene probe and target copy number. Scale bar = 5 μm. Exposure times (ms, milliseconds) are described for the gene images. The signal intensity increases with the increasing number of probes (higher exposure time was necessary when hybridizing with one probe). The signal spread and intensity increases with the increase in the target number, from dot-like for low target copy cells to whole cell signal for high target copy cells. For high target copy number cells, starting with ∼ 6 probes, the signal does not increase anymore with the probe number, most likely due to a saturation of tyramide binding sites. **Fig. S2.** Pseudoalteromonas sp. H100 growth curves based on triplicate measurements. The bacterial host was physiologically acclimated for three generations resulting in 0.72 doublings per hour (± 0.06 doublings per hour, n = 3) during exponential growth. Error bars indicate standard deviation. **Fig. S3.** Virus assays including controls. A. Extracellular phage DNA as measured by quantitative PCR in infected (black circles) and control (white circles) cultures. B. Extracellular phage number as measured by PFU in infected (black circles) and control (white circles) cultures. Control data are zero unless plotted otherwise. **Fig. S4.** Determination of phage signal size classes and segregation of the two waves of infection. A. Plot of signal size versus signal intensity for T0-T81. B. Plots of signal size versus signal intensity for each of the individual time points (from T0 to T81). Blue lines delimitate signal size classes. Class I (< 0.4 μm^2^): most probably new infections; Class II (0.4-1.4 μm^2^): most probably replicating infections; Class III (1.4-7.0 μm^2^): most probably advanced infections. To establish the upper and lower limits of the smallest, first size class, we assumed that T0 signals represented new infections - these signals have both a small area and a low intensity (panel B). To establish the bounds of the largest, third size class, the first time point where both signal area and intensity were maximum (T36) was considered to represent advanced infections, i.e. late replication and assembly. All signals between those two size classes were considered as size class II, that is replicating infections - for examples, compare T0 with T21 and T36. While at T36 there were almost no class I signals, at T51 they reappeared and were abundant at T66 and T81. Furthermore, the class III signals decreased in abundance at T66 and T81. The re-appearance of class I signals in T51-T81 was assumed to represent new infection events by newly released mature phage particles and thus, a second wave of infection. All other T51-T81 signals were considered old infections from the first wave, in the process of phage maturation and release. **Fig. S5.** Localization of encapsidated phage and host cell ribosomes in TEM image of phage-infected *Pseudoalteromonas* cells from T66. Magnification 40 000×, scale bar = 500 nm. **Fig. S6.** PhageFISH with the negative control gene probe (NonPoly350Pr) on infected cells from T81. The false positive events (white arrows) are all in the smallest signal size class and they amount to a background of ∼ 2% from the cells. No false positives similar to the signals in the higher size class ategories or to the cell bursts releasing phage particles are visible. **Fig. S7.** Reconstruction of the Alexa_594_ image T21 from Fig. 2A by using the High Dynamic Range Imaging protocol. A-C. Exposure time series. D. Reconstructed image. **Fig. S8.** Reconstruction of the Alexa_594_ image T36 from Fig. 2A by using the High Dynamic Range Imaging protocol. A-C. Exposure time series. D. Reconstructed image **Fig. S9.** Reconstruction of the Alexa_594_ image T51 from Fig. 2A by using the High Dynamic Range Imaging protocol. A-B. Exposure time series. C. Reconstructed image. **Fig. S10.** Reconstruction of the Alexa_594_ image T66 from Fig. 2B by using the High Dynamic Range Imaging protocol. A-D. Exposure time series. E. Reconstructed image. **Fig. S11.** Reconstruction of the Alexa_594_ image T81 from Fig. 2B by using the High Dynamic Range Imaging protocol. A-E. Exposure time series. F. Reconstructed image. **Fig. S12.** Reconstruction of the Alexa_594_ image T96 from Fig. 2B by using the High Dynamic Range Imaging protocol. A-D. Exposure time series. E. Reconstructed image. **Fig. S13.** Reconstruction of the Alexa_594_ image T111 from Fig. 2C by using the High Dynamic Range Imaging protocol. A-D. Exposure time series. E. Reconstructed image. **Fig. S14.** Reconstruction of the Alexa_594_ image T126 from Fig. 2C by using the High Dynamic Range Imaging protocol. A-E. Exposure time series. F. Reconstructed image. **Fig. S15.** Reconstruction of the Alexa_594_ image T141 from Fig. 2C by using the High Dynamic Range Imaging protocol. A-D. Exposure time series. E. Reconstructed image.
