# Supplementary material for: Single-cell and population level viral infection dynamics revealed by phageFISH, a method to visualize intracellular and free viruses
Source: Environ Microbiol. 2013 Mar 14;15(8):2306–18. doi: 10.1111/1462-2920.12100 (PMC3884771; doi:10.1111/1462-2920.12100)
Supplement: Supplementary file 1 — SI Text. Table S1. Polynucleotide probes targeting a region spanning a phage gene of unknown function (abbreviated here as unk) in the Pseudoalteromonas phage PSA-HP1 genome (genome position 8564-13 387 bp). Table S2. Q-PCR primer and amplicon sequences. Table S3. Calculated and measured Tm for the polynucleotides forming the unk probe mix. Fig. S1. geneFISH protocol optimization. A. Genome map of phage PSA-HP1. The six (300 bp each) probe-target regions are indicated in orange. Probes target unknown phage gene, unk (grey). B. Variation of the gene detection efficiency with increasing number of polynuclotide probes. Escherichia coli low target copy clones (3-8 copies per cell) were hybridized with an increasing number of polynucleotide probes targeting the unk gene. As negative control (no unk gene), E. coli strain B/R cells were used. The detection efficiency is defined as the fraction of all cells showing a gene positive signal. C. Detection of unk gene in high target copy cells using three polynucleotide probes - all the cells have a gene signal, resulting in 100% detection efficiency. Top image - overlay image between 16S rRNA signal and gene signal. Bottom image - gene signal. Scale bar = 5 μm. Exposure time (ms, milliseconds) is described for the gene image. D. Appearance of gene signals for different dextran sulfate concentrations (10%, 20% and 30%). All pictures were taken using the same exposure time. Concentrations of 20% and 30% dextran sulfate resulted in a much sharper signal as compared with 10%. Scale bar = 5 μm. E. Gene detection efficiency for different dextran sulfate concentrations (10%, 20% and 30%). Blue bars = low target gene copy cells, red bars = negative control cells. While the detection efficiency was high for all concentrations, the background level (% of false positives in the negative control) increased with the dextran sulfate concentration. F. Variation of the gene (unk) signal intensity and spread through the cell with variation of t [file emi0015-2306-sd1.pdf]

Single-cell and population level viral infection dynamics revealed by phageFISH, a method to visualize intracellular and free viruses

Elke Allers*, Cristina Moraru*, Melissa B. Duhaime, Erica Beneze, Natalie Solonenko, Jimena Barrero Canosa, Rudolf Amann**, Matthew B. Sullivan**

* Authors contributed equally to the manuscript

** Corresponding authors: rmann@mpi-bremen.de, mbsulli@email.arizona.edu

Supporting information

Table of contents

SI Text 3

Bacterial growth curves 3

One-step growth experiment 3

Enumeration of phage – SYBR stain 3

Enumeration of phage – quantitative PCR: qEXT 4

Enumeration of phage – Assay of plaque-forming units (PFU) 4

Enumeration of hosts – DAPI 4

Enumeration of infected host cells – FVIC 4

phageFISH 5

References 11

SI Tables 12

Table S1: Polynucleotide probes targeting a region spanning a phage gene of unknown function (abbreviated here as *unk*) in the *Pseudoalteromonas* phage PSA-HP1 genome (genome position 8564-13,387 bp). 12

Table S2: Q-PCR primer and amplicon sequences. 15

Table S3: Calculated and measured T_m for the polynucleotides forming the *unk* probe mix. 16

SI Figures 17

Figure S1: geneFISH protocol optimization. 17

Figure S2: Pseudoalteromonas sp. H100 growth curves 19

Figure S3: Virus assays including controls. 20

Figure S4: Determination of phage signal size classes and segregation of the two waves of infection 21

Figure S5: Localization of encapsidated phage and host cell ribosomes in TEM image of phage-infected Pseudoalteromonas cells from T66. 23

Figure S6: PhageFISH with the negative control gene probe (NonPoly350Pr) on infected cells from T81 24

SI Appendix A 25

SI Text

Bacterial growth curves

The host, marine *Pseudoalteromonas* sp. str. H100 (Wichels *et al.*, 1998; kindly provided by A. Wichels, AWI Helgoland), was grown in 50 ml cultures in 20% nutrient Zobell marine media (0.2 g yeast extract [BD, cat. no. 212750], 1 g proteose peptone [BD, cat. no 211684], and 26 g sea salts [26 PSU; Sigma-Aldrich, cat. noS9883] per liter, pH 7.6) on an orbital shaker (130 rpm, 21°C) for 24 hours. Each day, the culture was transferred 1:10 to fresh media. Growth curves and rates were monitored for three consecutive generations to ensure stable growth before the start of the one-step growth experiment (below). The optical density was measured in triplicate using the Appliskan Multimode Microplate Reader (Thermo Scientific, Rockford, IL, USA). DAPI counts (described below) were obtained at 5 points along the growth curve in order to correlate OD with cell count to reach a targeted multiplicity of infection (MOI) accurately for the one-step experiment.

One-step growth experiment

In order to gauge the success of the phageFISH procedure, infection dynamics of the *Pseudoalteromonas* sp. H100 and podovirus PSA-HP1 (Wichels *et al.*, 1998; kindly provided by A. Wichels, AWI Helgoland) system were monitored through a one-step growth experiment. Through highly resolved sampling, we tracked the infection from phage entry to the assembly of new phage particles such that the phageFISH observations could be confirmed through a combination of qPCR, plaque assays and electron microscopy.

For the one-step growth experiment, the H100 host was grown overnight in 20% nutrient Zobell marine media, as above, and transferred 1:10 to a new 60 ml culture. After 3 hours of growth (10^8 cells ml^{-1}; early exponential phase; Fig. S2), phage PSA-HP1 was introduced at an MOI of 0.5 in duplicate. Marine SM buffer (100 mM NaCl, 81.2 mM $MgSO_4 \cdot 7H2O$, 50 mM Tris-HCl [pH7.5]), the buffer in which the phage lysates are stored, was added to two negative controls. Phages were allowed to adsorb to host for 19 min, and then 7 ml of all treatments and controls was diluted 1:100 with 20% nutrient Zobell marine media to prevent additional adsorption, thus synchronizing the infection across the population. At this point (T0), sampling began every 15 min for qPCR, plaque assays, DAPI counts, FVIC and phageFISH (all described below). At each time point, sampling for all parameters was performed consistently within 5 min, except T0, which took 10 min.

In the broader context of one-step growth experiments, we point out one apparent inconsistency in the present experiment. Based on predictions assuming a Poisson distribution of phage-host encounters, whereby percent infected cells is predicted to equal $1-e^{-MOI}$, our experimental MOI (0.5) should have resulted in 36% of cells infected by one phage (Knipe *et al.,* 2001). However, both phageFISH and FVIC suggest that <20% of cells were infected. Given the methodological concordance, this fraction of infected cells appears to be a biological reality and implicates another cause for the reduced fraction of cells infected. We posit that the difference stems from the simplicity of the MOI based formula, which, when calculating the fraction of infected cells, does not take into account factors like cell concentration or time allowed for adsorption.

Enumeration of phage – SYBR stain

One µl of PSA-HP1 phage lysate was stained in 500 µl TE (10 mM Tris-HCl, 1mM EDTA, pH 7.5) with 1 µl of 10,000X SYBR Gold (Invitrogen, cat. no. S11491, Grand Island,

NY), diluted 1:20 in TE for 12—15 minutes, then filtered onto an Anodisc 0.02 µm 25 mm filter (Whatman, cat. no. WHA-6809-6002). Stained phages were counted on an AxioImager microscope (Carl Zeiss, Germany). A total of >900 phages were counted over 30 fields of vision.

Enumeration of phage – quantitative PCR: qEXT

To track phage PSA-HP1's infection parameters (e.g., burst size, latent period), a qPCR assay to quantify extracellular viruses (qEXT) was designed according to Lindell and colleagues (2007). Primers (PSA-HP1_dF, PSA-HP1_dR; Table S2) were designed to quantify a single copy, non-coding sequence motif (30 bp) from the PSA-HP1 phage genome. Q-EXT was determined by quantifying the PSA-HP1 target sequence present in the 0.2 µm sample filtrate. Q-PCR (Eco Real-Time system; Illumina Inc., San Diego, CA) was performed with the 2X QuantiTect SYBR Green kit (Qiagen, cat. no. 204143, Valencia, CA) in 25 µl triplicate reactions (control samples were performed in 12.5 µl triplicate reactions) with primers at a final concentration of 0.3 µM, followed by an 80-cycle melt-curve analysis: 95 °C, 15 min; 55x (94 °C, 15 sec; 61°C, 30 sec; 72 °C, 30 sec); 90x (50 °C, 15 sec) increasing 0.5 °C with each cycle. A dilution series of the phage stock ranging from ten to one million copies per reaction was used in triplicate as a standard for each qPCR run.

Enumeration of phage – Assay of plaque-forming units (PFU)

To verify the qPCR-based phage quantification of extracellular phage (qEXT), plaque assays were performed on the same set of samples using the "agar overlay" method. Briefly, a solid agar base (10 ml of 1.2% agar, w/v) was overlaid with a mixture of soft agar (3 ml of 0.6% agar, w/v), 400 µl overnight-grown H100 culture, and 100 µl of diluted sample from the one-step growth experiment, such that plaques representing viral infection form in the soft agar overlay. Serial dilutions of the one-step growth experiment samples were plated in triplicate and incubated at room temperature overnight and plaques on plates of the appropriate dilution were numerated.

Enumeration of hosts – DAPI

Cells were immobilized on 25 mm 0.2 µm GTTP filter (Millipore, cat. no. GTTP02500) using vacuum filtration. Samples were embedded in a mix of Vectashield and Citifluor (1:4) containing 1 µg ml^{-1} 4′,6-diamidino-2-phenylindole (DAPI). At least 1000 cells were counted at 1000x magnification over at least 12 different fields of vision using an AxioImager microscope (Carl Zeiss, Germany).

Enumeration of infected host cells – FVIC

For a count of the frequency of visibly infected cells (FVIC, Proctor *et al.*, 1993), 10 ml samples were fixed with 2% EM-grade formaldehyde, flash frozen in liquid nitrogen and stored at -80 °C until processing. After thawing, cells were pelleted and washed in 0.1 M PIPES buffer (pH 7.4), post-fixed in 2.5% glutaraldehyde for 30 min, washed with 3 changes of PIPES and further fixed in 1% osmium tetroxide for 15 min. Pellets were washed twice in deionized water and stained with 2% aqueous uranyl acetate. Following dehydration through an alcohol series, pelleted cells were washed in 3 changes of propylene oxide and infiltrated with a 1:1 mix of propylene oxide and Embed 812 resin overnight. Following infiltration with 3 changes of resin for 60 min each, samples were polymerized in fresh resin overnight at 60 °C. Seventy nm sections were cut onto 150 mesh uncoated copper grids, post-stained with 2% lead citrate and viewed in an FEI CM12S electron microscope, operated at 100 kV. For each time point, the first 800 intact cells from one of the biological

replicates were examined at 32,000–88,000 magnification. If the number of mature viruses in a cell was >5, it was scored as infected (Brum *et al.*, 2005). Eight bit TIFF digital images were captured by an AMT420 camera.

phageFISH

***Escherichia coli* cultures for method optimization**

The low and high target copy cultures of the *unk* clone contained the *unk* gene in different copy numbers (low target copy clone: 3–8 copies, high target copy clone: up to 200 copies), while *E. coli* B/R had no *unk* gene and was used as negative control. To obtain the *unk* clone, the *unk* gene of phage PSA-HP1 was cloned into a pCC1 vector and transformed in *E. coli* Epi300 cells using the Copy Control cDNA, Gene and PCR Cloning Kit (Epicentre, cat. no. CCPCR1CC) according to the manufacturer's instructions. The different *unk* copy number cultures were obtained according to Wild *et al.* (2002) as follows. The *unk* clone was first grown over night in LB media (1% Tryptone, 0.5% Yeast Extract and 1% NaCl) with 12.5 µg/ml Chloramphenicol, starting from single colonies. Then it was inoculated in fresh media containing 0.2% glucose (low target copy culture) or 1x Induction Solution (Epicentre, cat. no. CCIS125, high target copy culture). *E. coli* B/R was inoculated in LB media. All cultures were grown at 37 °C and 200 rpm and cells harvested in mid-log phase. For the low copy culture, the plasmid copy numbers were confirmed according to Bremer and Dennis (1996) and Meyenburg and Hansen (1996), as follows: the growth rate (2.8 doublings per hour) was used to calculate the number of genome equivalents per cell (3.7 genome equivalents), which was further used to confirm the number of plasmid copies (1-2 copies per genome equivalent, therefore 3-8 plasmid copies per cell). For the high copy culture, the induction was confirmed by agarose gel electrophoresis. The cells were fixed by adding paraformaldehyde (Electron Microscopy Sciences, cat. no. RT 15713) to a final concentration of 2%, followed by overnight incubation at 4 °C. To wash and concentrate the cells, the fixed cultures were filtered through 0.2 µm polycarbonate membrane filters (GTTP, Millipore, cat. no. GTTP02500) mounted in Swinnex filter holders (Millipore) and washed with 50 ml of 1x PBS. After recovery of the cell suspension, 96% ethanol was added in 1:1 ratio and the cells were stored at -20 °C.

Probe design, probe synthesis and hybridization stringency

To target the *unk* phage gene, 12 dsDNA polynucleotide probes (300 bp each) were prepared. The sequence and the target region for each probe are given in Table S1. Of these 12 probes, only the first 6 (see Fig. S1A) were used in the one-step growth experiment, while all 12 were used in the optimization experiments on *E. coli* (Fig. S1B). NonPoly350Probe (Moraru *et al.*, 2010) was used as gene negative control for the one-step growth experiment samples. Probes (with Dig) were produced by incorporating Dig-dUTP into dsDNA via PCR (70 µM Dig-dUTP), using the PCR Dig Probe Synthesis Kit (Roche, cat. no. 11636090910), according to the manufacturer's instructions. Targets (without Dig) for the melting temperature determinations were produced similarly with the probes, but without Dig-UTP in the PCR mix. The PCR products were column purified using the Gene Clean Turbo kit (Q-Biogene, cat. no. 1102-600), eluted in water, and checked electrophoretically in 2.5–3% agarose gels, for the right size and for Dig incorporation. The probes positioned themselves in the gel at a higher position than their target counterparts, a clear indication of Dig incorporation. The concentration was determined using a Quant-iT^{TM}Picogreen assay

(Invitrogen, cat. no. P7589) and spectrophotometrically, using a NanoDrop 1000 Spectrometer (Thermo Scientific). The probes were stored at -20 °C.

The conditions for hybridization were determined as described by Moraru and colleagues (2010). Firstly, the melting temperature (T_m) for each probe–target pair was calculated using the PolyPro software (Moraru *et al.*, 2011). Secondly, the T_ms of the probe–target hybrids were measured in a buffer with a similar composition to that of the hybridization buffer, using the Eco™ Real-Time PCR system (Illumina, San Diego, CA, USA) and SYTO 9 dye (Invitrogen, cat. no. S-34854). The hybridization-like buffer was composed of 1.75 ml formamide (Sigma), 1.25 ml 20x SSC (3 M NaCl, 0.3 M sodium citrate), 0.5 g dextran sulfate sodium salt (Sigma, cat. no. D8906), 25 µl 20% sodium dodecyl sulfate (SDS), 0.2 ml 0.5 M EDTA pH 8.0 and 1.3 ml autoclaved MilliQ water. To 1.5 ml of hybridization-like buffer, 3 µl of 5 mM SYTO 9 were added for a final concentration of 10 µM. To 100 µl of the latter mixture, 6 µl of dsDNA (230-350 ng) were added, and the resulting solution was aliquoted into 25 µl portions per well and used for T_m determinations. The T_m was measured for the probe dsDNA (both strands with Dig-dUTP), for target dsDNA (both strands without Dig-UTP) and for a mixture of the probe and the target, which, during the hybridization phase of the thermal protocol, resulted in a hybrid dsDNA (one strand with Dig-UTP and the other without). The thermal protocol used for the T_m determination in hybridization-buffer like buffer was the following: denaturation at 85 °C for 5 min, hybridization at 44 °C for 25 min and melting from 50 °C to 80 °C, at 5.5 °C per sec average ramp rate. Based on the T_m values, the hybridization parameters for the *unk* probe mix were determined (see Table S3) as detailed in (Moraru *et al.*, 2010; Moraru *et al.*, 2011): denaturation temperature at 85 °C, hybridization at 42 °C and washing at 42 °C.

PhageFISH protocol for (i) combined phage gene detection with rRNA detection for the identification of host cells, and (ii) detection of free phage particles

<u>General considerations</u>

The water used during the procedure was autoclaved 0.22 µm filtered MilliQ water. Unless stated otherwise, the incubations were performed at room temperature (RT). All washing steps were carried out in 50 ml volumes, in plastic Petri dishes when the steps were performed at room temperature or in the oven, or in 50 ml Falcon tubes, when incubation in water bath was necessary.

Buffers containing dextran sulfate (DS) were prepared by first dissolving at 48 °C the DS (Sigma, cat. no. D8906) in the buffer components, with the exception of formamide, SDS and blocking reagents. When the DS was completely dissolved, the solution was cooled to room temperature and the rest of the components were added. After preparation, the formamide-containing buffers were stored at -20 °C, while the formamide-free buffers were filtered sterilized (0.2 µm) and kept at 4 °C.

The $Alexa_{488}$ and $Alexa_{594}$tyramides were prepared as described by Pernthaler and Pernthaler (2005).

At any time during the procedure, unless specifically indicated in the protocol, the samples were not allowed to dry. Drying was especially dangerous during hybridization or CARD reactions, when it could cause background formation. To avoid drying, all the incubations were done by completely immersing the filters in the respective buffers. When smaller volumes of buffers were used and the incubation was done at a higher temperature and/or for a longer time, the samples were placed in humidity chambers. A humidity

chamber can be any tightly closing container that seals with a silicone O-ring. For low temperature incubations (e.g. room temperature, 37 °C, 46 °C), polypropylene containers were used. However, for high temperature incubations (e.g. 85 °C), containers made of glass (lid can be of polypropylene) were used to avoid deformation of the container and drying of the samples. To create humidity in the chamber, the bottom was lined with tissue paper soaked in water or, when the buffers contained formamide or paraformaldehyde, in a formamide-water or paraformaldehyde-water solution of the same concentration as the buffer. For samples immobilized on filters (*E. coli* and one-step growth experiment samples), the filters were placed face-up in Petri dishes, covered with buffer, and then the Petri dishes were placed in humidity chambers. For samples immobilized on slides (phage lysates), the sample area (marked with a glass pen) was covered with buffer and the slide placed in a humidity chamber (usually on top of a PCR tube rack).

Horseradish peroxidase (HRP) labeled oligonucleotides or antibodies, after being dissolved in water, were stored at 4 °C for no longer than 6 months. In both cases, vortexing was avoided and mixing was performed by pipetting up and down or gently inverting the tubes.

The stocks of fluorochrome labeled chemicals were stored in the dark. Excessive light exposure during the procedure was avoided.

The antibody step promotes the formation of false positives. These are more likely to appear when the cells are damaged during the phageFISH procedure, particularly during the acid treatments (necessary for the inactivation of the HRP, endogenous and introduced with the rRNA targeting probes) and denaturation step. Therefore, the strength of permeabilization, inactivation of peroxidases (by acid treatments or otherwise) and denaturation time must be carefully optimized to minimize damage to cells.

<u>phageFISH on *E. coli* clone cells and *Pseudoaltermonas* (one-step growth experiment) samples</u>

Sample immobilization. Different volumes of fixed *E. coli* cells were mixed with 10 ml 1x PBS and filtered through 0.2 µm polycarbonate filters (GTTP, Millipore, cat. no. GTTP02500). The *Pseudoalteromonas* samples were filtered directly after paraformaldehyde fixation. The filters were then washed with 15 ml water, air dried and stored at -20 °C.

Permeabilization. Permeabilization was undertaken by overlaying the filters with permeabilization solution – 0.5 mg ml^{-1} lysozyme (AppliChem, cat. no. A4972.0010), 1x PBS pH 7.4 (1370 mM NaCl, 27 mM KCl, 80 mM Na_2HPO_4, and 20 mM KH_2PO_4), 0.1 M Tris-HCl pH 8.0 and 0.05 M EDTA pH 8.0, for one hour, on ice. The washes consisted of 5 min with 1x PBS and 1 min with water.

Inactivation of endogenous peroxidases. Inactivation was performed by immersing the filters in 0.01M HCl for 10 min, followed by washing with 1x PBS for 5 min, water for 1 min and 96% ethanol for 1 min, followed by air-drying. To evaluate the efficiency of endogenous peroxidases inactivation, *a priori* tests were performed as follows. *Pseudoalteromonas* cells were hybridized with the positive control EUB338 probe and with the negative control NON338 probe, during a complete rRNA CARD-FISH protocol. This protocol consisted from sample immobilization, permeabilization, inactivation of endogenous peroxidases, rRNA hybridization, CARD for rRNA detection and embedding and counterstaining, all performed as described in this phageFISH protocol. Microscopic evaluation revealed no signals in the negative control and FISH signals in the positive control, therefore confirming the efficiency of the endogenous peroxidases procedure.

rRNA hybridization. The HRP-labeled EUB338 probe (Amann *et al.*, 1990), synthesized by Biomers (Ulm, Germany), was added to a final concetration of 0.16 ng µl^{-1} to hybridization buffer containing 35% formamide. Mixing was performed by gentle shaking, no vortexing, to avoid the removal of the HRP from the oligonucleotide. The filtered samples were covered with hybridization mixture and placed in a humid (35% formamide solution) chamber. Hybridization took place for 3 h at 46 °C, followed by 15 min of washing at 48 °C. The hybridization buffer contained 35% formamide, 10% dextran sulfate, 0.9 M NaCl, 20 mM Tris-HCl, 1% blocking reagent (Roche, Germany, cat. no. 11096176001), 0.25 mg/ml sheared salmon sperm DNA (Ambion, cat. no. AM 9680), 0.25 mg/ml yeast RNA (Ambion, cat. no. AM 7118) and 0.02% SDS. The washing buffer contained 70 mM NaCl, 5 mM EDTA pH 8, 20 mM Tris-HCl and 0.1% SDS.

CARD for rRNA detection. All the samples were equilibrated for 15 min in 1x PBS. The samples were incubated for 10 min at 37 °C in a solution containing 1x PBS, 10% dextran sulfate, 0.1% blocking reagent (Roche, Germany, cat. no. 11096176001), 2 M NaCl, 0.0015% H_2O_2 and 0.33µg ml^{-1} Alexa$_{488}$-labeled tyramides. The wash steps were 10 min with 1x PBS at 46 °C, 1 min with water and 1 min with 96% ethanol, followed by air-drying.

RNase treatment. The samples were overlaid with RNase solution (0.1 U µl^{-1} RNase I [Ambion, cat. no. AM 2295], 75 µg ml^{-1} RNase A [Sigma, cat. no. R4642-10], 0.1 M Tris-HCl, pH 8.0) and incubated overnight at 37 °C. The washes consisted of 2x 5 min with 1x PBS and 1 min with water.

Inactivation of HRP introduced with the rRNA probe. The inactivation of HRP consisted of 10-min incubation in 0.2 M HCl, followed by washing with 1x PBS for 1 and 5 min, then 1 min with water, 1 min with 96% ethanol and air-drying.

Gene hybridization. Prehybridization was carried out for 3.5 h at 42°C by overlaying the samples with the same buffer as for hybridization, but without the probe. The hybridization buffer had the following composition: 1.75 ml formamide, 1.25 ml 20x SSC, 0.5 g dextran sulfate, 25 µl 20% SDS, 0.2 ml 0.5M EDTA pH 8.0, 0.55 ml water, 125 µl sheared salmon sperm DNA (sssDNA; Ambion, cat. no. AM 9680), 125 µl yeast RNA (Ambion, cat. no. AM 7118) and 0.5 ml 10% blocking reagent (Roche, Germany, cat. no. 11096176001). For hybridization, the samples were transferred into probe containing hybridization buffer. *E.* coli samples were hybridized with hybridization mixtures containing 1 up to 12 *unk* probes, while *Pseudoalteromonas* samples were hybridized with 6 probes (Pr*unk1-6* – see Table S1.) Each probe was added to a final concentration of 5 pg µl^{-1}. The NonPoly350Pr was added to a final concentration of 30 pg µl^{-1} (the equivalent of 6 *unk* probes). The samples were first denatured – *E. coli* for 1h, *Pseudoalteromonas* for 35 min or 1 hour - in an 85 °C hybridization oven. Both incubation times gave the same number of phage infected cells for size classes II and III, however, 35 min underestimated the number of infected cells from size class I. On the other hand, the cells looked more damaged after 1 hour denaturation. After denaturation, the tubes were transferred immediately to a 42 °C oven and hybridization took place for 18–22 h. The washes were first performed with washing buffer I (WBI; 2x SSC, 0.1% SDS) 3x for 1 min at RT and 30 min at 42 °C, followed by washing buffer II (WBII; 0.1X SSC, 0.1% SDS) 3x for 1 min at RT and 1.5 h at 42 °C in a slow shaking water bath, and finally 1 min with 1x PBS.

Antibody binding. The samples were incubated in a solution of 1x PBS and 1% Western Blocking Reagent (WBR; Roche, cat. no. 11921673001) for 45 min. The antibody binding took place for 1.5 h, in a solution containing 1x PBS, 1% WBR and 0.3 U ml^{-1} (500x dilution of the 150 U/ml stock) anti-Dig HRP-conjugated antibody (Fab fragments; Roche,

cat. no. 11207733910). The wash was carried out in a solution of 1x PBS and 1% WBR solution for 1 min and 3x 10 min. All steps were carried out on a shaker at 20 rpm.

CARD for gene detection. The samples were overlaid in amplification buffer containing 1x PBS, 20% dextran sulfate, 0.1% blocking reagent, and 2 M NaCl with 0.0015% H_2O_2 and 2 µg ml^{-1} $Alexa_{594}$-labeled tyramide and incubated for 45 min at 37 °C. They were then washed for 1 min, 5 min and 2x 10 min with 1x PBS in a 46 °C oven, slow shaking, then 1 min with water, 1 min with 96% ethanol, followed by air-drying. To find the best amplification buffer, different dextran sulfate concentrations (10%, 20% and 30%) were tested on *E. coli* clones prior to evaluating one-step growth experiment samples.

Embedding and counterstaining. The samples were embedded either in ProLong Gold antifade reagent (Invitrogen, cat. no. P36930) or SlowFade Gold antifade reagent (Invitrogen, cat. no. S36936) containing 1 µg ml^{-1}4´,6-diamidino-2-phenylindole (DAPI) and stored at -20 °C.

phageFISH on phage lysate samples

Sample immobilization. A volume of 100 µl phage lysate was spotted on PolyLysine glass slides (ThermoScientific, cat. no. J2800AMNZ, pre-cleaned with ethanol and sample area marked on the back with a Glascribe Pen from Electron Microscopy Sciences) and air-dried at 37 °C for 50 min. To remove salts, a 1 min wash was done in water, followed by 1 min in 96% ethanol and air-drying.

Fixation. The sample area was overlaid with a 1% PFA solution and incubated for 1 hour at room temperature. Washings consisted of 5 min 1x PBS, 1 min water, 1 min 96% ethanol, followed by air-drying.

HCl treatments to open the viral capsid. Samples were incubated in 0.01 M HCl for 10 min and 0.2 M HCl for 10 min, followed by washings – 1 and 5 min 1x PBS, 1 min water, 4 min 96% ethanol, air-drying.

Gene detection. From here on, the same procedure as for the one-step infection samples was followed, starting with gene hybridization.

Embedding and counterstaining. The samples were embedded in SYBR Green-containing mounting media (Lunau *et al.*, 2005).

Microscopy, cell counts and image processing of phageFISH samples

Microscopy was performed on an Axioskop2 Mot Plus epifluorescence microscope (Carl Zeiss, Germany), equipped with the following fluorescence filter sets: $Alexa_{488}$ (472/30 excitation, 520/35 emission, 495 Beam Splitter) and $Alexa_{594}$ (562/40 excitation, 624/40 emission, 593 Beam Splitter). The $Alexa_{488}$ filter set was used for detection of the 16S rRNA signals, while the $Alexa_{594}$ filter set was used for detection of the phage gene signals. Both for cell counts and image processing, photomicrographs were taken with a black and white digital camera, AxioCamMn (Carl Zeiss, Germany), using the AxioVision 4.8 software (Carl Zeiss, Germany). To capture both the strong, cell-wide and the weak, dot-like phage signals, a series of images with increasing exposure times (e.g. 3 ms, 5 ms, 7 ms, 10 ms, 15 ms, 25 ms, 40 ms, 50 ms, 75 ms, 100 ms and 140 ms) was taken for the $Alexa_{594}$ filter set. The thresholds for a phageFISH positive signal were: signal area ≥ 0.06 µm2 and signal intensity ≥ 30 gray value, in a 10 ms exposure image. The black and white photomicrographs were pseudo-colored automatically by the software used for acquisition, green for the 16S rRNA and red for the phage signals.

Counting was performed on photomicrographs, by manually marking the cells in the $Alexa_{488}$ channel and the corresponding gene signals in the $Alexa_{594}$ channel with the “Events” tool from the “Measure” menu. The number of events was determined using the “measure events” function. At least 800 cells were counted per sample and the standard deviation was calculated from the biological replicates (*Pseudoalteromonas*) or from technical triplicates (*E. coli*). For the one-step experiment, the number of infected cells was corrected for the number of false positives (2.1% ± 0.4%) found in the negative control (infected cells hybridized with the NonPoly350Pr probe). This correction applied only to the signals from the lower size class (<0.4 μm). The size of the phage signals was measured by marking the signals with the free hand tool in Zen Lite 2011(Blue Edition; Carl Zeiss, Germany) software. The images acquired with 10 ms exposure time were used and ~100 cells were analyzed for each time point.

Image processing was performed first by using the Zen Lite 2011 software (Blue edition; Carl Zeiss, Germany) to export the selected fields of view and then by using the PaintShop Photo Pro X4 (Corel Corporation, USA) to reconstruct the images from the exposure time series (see below).

The signals in the $Alexa_{594}$ filter set were characterized by different sizes and intensities, in such a way that the exposure times at which the large signals were not overexposed did not allow the small signals to be visible, while the exposure times at which the small signals were visible resulted in a serious overexposure of the large signals. To reconstruct the image, we devised a High Dynamic Range Imaging protocol. Accordingly, images with increasing exposure times were loaded as separate layers. First, different elements composing an image were identified. Then, for each element, the layer where the element was clearly visible, but not overexposed, was selected. The elements were merged into one new image, by transferring the information from the higher exposure layers to the lower exposure layers, using the Eraser tool. At the end, a sharpening filter and a black threshold were applied on the reconstructed images. For each reconstructed image, the original images of the exposure time series, as well as the reconstructed image, are shown in Figures S7 to S15 in SI Appendix A. The overlay between the green 16S rRNA signals and the red phage signals was performed by visualizing the layers with the “Lighten” function.

<u>Calculation of detection efficiencies with increasing number of probes</u>

To calculate the detection efficiencies with increasing probe numbers, formulas taking into account the probability (P) of more than one probe binding have been applied. The formulas employ the probability that the binding of the probes is not mutually exclusive, which can be solved by: [the probability that the binding is mutually exclusive (e.g. for two probes this is $P(A) + P(B) = 2 \times P(A)$)] – [the probability that the binding is independent (e.g., for two probes this is $P(A) \times P(B) = P(A)^2$)].

For two probes, the formula will be: $P(2\ probes) = 2 \times P(A) - P(A)^2$

For three probes, the formula will be: $P(3\ probes) = 3 \times P(A) - C^3_2 \times P(A)^2 + P(A)^3$

For four probes, the formula will be: $P(4\ probes) = 4 \times P(A) - C^4_2 \times P(A)^2 + C^4_3 \times P(A)^3 - P(A)^4$

For 12 probes, the formula will be: $P(12\ probes) = 12 \times P(A) - C^{12}_2 \times P(A)^2 + C^{12}_3 \times P(A)^3 - C^{12}_4 \times P(A)^4 + C^{12}_5 \times P(A)^5 - C^{12}_6 \times P(A)^6 + C^{12}_7 \times P(A)^7 - C^{12}_8 \times P(A)^8 + C^{12}_9 \times P(A)^9 - C^{12}_{10} \times P(A)^{10} + C^{12}_{11} \times P(A)^{11} - P(A)^{12}$

$P(A)$ is the probability determined experimentally for 1 probe, and C^x_y is the number of combinations of y elements from a larger set of x elements.

References

Amann, R., Binder, B.J., Olson, R.J., Chisholm, S.W., Devereux, R., and Stahl, D.A. (1990) Combination of 16S rRNA-targeted oligonucleotide probes with flow cytometry for analyzing mixed microbial populations. *Appl Environ Microb* **56**: 1919-1925.

Bremer, H., and Dennis, P.P. (1996) Modulation of chemical composition and other parameters of the cell by growth rate. In *Escherichia coli and Salmonella*. Neidhardt, F.C., III, R.C., Ingraham, J.L., Lin, E.C.C., Low, K.B., Magasanik, B. et al. (eds). Washington D.C.: ASM Press, pp. 1553-1569.

Brum, J.R., Steward, G.F., Jiang, S.C., and Jellison, R. (2005) Spatial and temporal variability of prokaryotes, viruses, and viral infections of prokaryotes in an alkaline, hypersaline lake. *Aquat Microb Ecol* **41**: 247-260.

Knipe, D.M., Howley, P.M., and Griffin, D.E. (2001) *Field's virology*: Lippincott Williams & Wilkins.

Lindell, D., Jaffe, J.D., Coleman, M.L., Futschik, M.E., Axmann, I.M., Rector, T. *et al.* (2007) Genome-wide expression dynamics of a marine virus and host reveal features of co-evolution. *Nature* **449**: 83-86.

Lunau, M., Lemke, A., Walther, K., Martens-Habbena, W., and Simon, M. (2005) An improved method for counting bacteria from sediments and turbid environments by epifluorescence microscopy. *Environ Microbiol* **7**: 961-968.

Meyenburg, K.V., and Hansen, F. (1996) Regulation of chromosome replication. In *Escherichia coli and Salmonella typhimurium: cellular and molecular biology*. Neidhardt, F.C. (ed). Washington: ASM Press.

Moraru, C., Moraru, G., Fuchs, B.M., and Amann, R. (2011) Concepts and software for a rational design of polynucleotide probes. *Environ Microbiol Rep* **3**: 69-78.

Moraru, C., Lam, P., Fuchs, B.M., Kuypers, M.M.M., and Amann, R. (2010) GeneFISH – an *in situ* technique for linking gene presence and cell identity in environmental microorganisms. *Environ Microbiol* **12**: 3057-3073.

Pernthaler, A., and Pernthaler, J. (2005) Simultaneous fluorescence *in situ* hybridization of mRNA and rRNA for the detection of gene expression in environmental microbes. *Methods Enzymol* **397**: 351-371.

Proctor, L.M., Okubo, A., and Fuhrman, J.A. (1993) Calibrating estimates of phage-induced mortality in marine bacteria: Ultrastructural studies of marine bacteriophage development from one-step growth experiments. *Microb Ecol* **25**: 161-182.

Wichels, A., Biel, S.S., Gelderblom, H.R., Brinkhoff, T., Muyzer, G., and Schuett, C. (1998) Bacteriophage diversity in the North Sea. *Appl Environ Microb* **64**: 4128-4133.

Wild, J., Hradecna, Z., and Szybalski, W. (2002) Conditionally amplifiable BACs: Switching from single-copy to high-copy vectors and genomic clones. *Genome Res* **12**: 1434-1444.

SI Tables

Table S1: Polynucleotide probes targeting a region spanning a phage gene of unknown function (abbreviated here as *unk*) in the *Pseudoalteromonas* phage PSA-HP1 genome (genome position 8564-13,387 bp).

Probe name	Region covered	%GC	PCR primers
>PR*unk*-1	12533-12832c	43.7	1F: ggacagattgttggtac; 1R: atcatctacagcagcaag*
ggacagattgttggtactcgtatgttccagactgaagtcttagagaaagtaggtgttcatggtatatcagcgggggattatatcttcaaggctgatgacgtggatgctttatataactacgtacaag<br>acgctagtacttcagaagaacagatagaacgtgtagaagaaatcgtacagattatctcagatactcagtctccgttcggagagaacgtactgtaccgtactgacttcttaactaacttacgtgct<br>gagctagaacgagaaggcggtggtacgcagcttgctgctgtagatgat			
>PR*unk*-2	12233-12532 c	45.0	2F: gtactagatgctacagag; 2R:ttcaccgttagcgtccg*
gtactagatgctacagaggtaggtttcctagctaagacattaacaggattccttaaggcatcaggggctaagctgttcggttcagctgctgctgctaataagacgttaagttcagaagatgctga<br>ctacatacgttcagtaatgcaagaagctgctgactctttatctcctagtaacggtatagcccataaacctcagatggttactgtagagaacaagggttccttgcttgataccctagcagttaataa<br>accagagaccctgcgtactgctcttaatgcagcggacgctaacggtgaa			
>PR*unk*-3	11933-12232 c	44.6	3F: gatgttatgcagtacctag; 3R: ttcccaagaagactgag*
gatgttatgcagtacctaggtgtaactaagtcaggcgtagcttctcgactaacacctactggtacagctgactcagggttgagctatacagctaccgagaaacgtcataagcttgaccagatat<br>cagagttcgactacagctctcttgataagactcgtgaagagttcgaacgtctagtatctaatcagaacgttagtgatatacttactcaggaagaacttgagtcaggtgttgaacgtagtatccaa<br>actatctacgctcagtcacgtgctacactgcaccctcagtcttcttgggaa			
>PR*unk*-4	11633-11932 c	42.6	4F: gcattagagaagaacgg; 4R: gtaagaatgagcaggtaa*
gcattagagaagaacggtgacttccttaaagctcgtgctatcttcggtaaggatggtacgtcagggttcgatacacttaaggatgctgaggtagctaagcgtaacttattcgtagaagggaag<br>atatctgcccgtgttgctggagagaacagtgcattaaggtctctagatgaggttaatgatataccagaagacaaccttgagtacttcattgatgttgaagtagagcatcaggttacttctaaggac<br>gttaaccctctagaaggtaaggtagatagttacttacctgctcattcttac			
>PR*unk*-5	11333-11632 c	43.0	5F: ttaatacctttcagctc; 5R: agccttattacgtataat*
ttaatacctttcagctcacgggtagagaagtcagtagttgactcagctatggcttatacggataaagcttcacgtatcgctgttactatgcaggactttactaagcctattgggaagttacgtggta			

agatggagcgcgaacagtggactaggcttcttactcatggggatgagcacggtattacctttaataaggtagctgctaaggaatacttaggtaagatgtccgatagagtatgggatgcgtacgtaggtacacgttctttctatgatggtatggctattatacgtaataaggct			
>PR*unk*-6	11033-11332 c	44.6	6F: caccgttcaggtctatc; 6R: tactatactgaagtcaa*
caccgttcaggtctatcagagcgtggattccgtggtatcaaggatgggattaatacattagaggattctaaagggaagttattcggtatacctatggcagaacgtccgttcattgtccctgaggcagccgctgacagccttactcaagttaatggtacaaagatatacaacagtgttactggtacagcagagacgcttacagacgacctattagatgctgcttatgcagagggtaatatcgtagtacgtactcatcgccctcttaattcaggtggtgagctgtttgacttcagtatagta			
>PR*unk*-7	10733-11032	46	7F: cgtcgtgatacagtacaa; 7R: tactgcttcgcgagaag*
cgtcgtgatacagtacaagacttacctcatcagactatgaacatccgtaagggtcacgtagaccttaactacctaggagaggactcgttcttctctaagtacatagctaagataggtggtggtggtcactcaggtggcacaggtgcgaagcttatccgtaaaggtaacgtaatacgtaacggagtaaagaccgagtcaactgaagtaatcggtatctaccgtgatactaagcaagctgctcaagctgctgaggatttaggagaggaaggtcttgaagttatctcttctcgcgaagcagta			
>PR*unk*-8	10433-10732	45.3	8F: tctgaactaggtgttgat; 8R: tataagcttctgtatctc*
tctgaactaggtgttgattcagtaggtactagctctaacgtaccgtctcatgcacgaggaagaggacaacgtctactaggcccatcaggttacgcagagatatctgacgtagaggacagtattggacgtgctcttggtgaagctagacgctatacaggtatggattcagttaacgtacttaagtctaagttcatggctacttacggtaagcatctaggtaacgctaaggaaggattccctactgacttcagtcgtactcagtggagaggtaaggataagccagagatacagaagcttata			
>PR*unk*-9	10133-10432	44.7	9F: cgggatgctaaagcttat; 9R:actagctgctcgtacag†
cgggatgctaaagcttatcatggatatattaagaaccaagaggctgcattaactggtacattacagactaagttccttgaagacttacgttcattcgtaggagcatggagagtagaaggtaatcagttccaagagttcattggtgacgcaggtgtatcgttacttgataaggatttcgttaaggtaggcactaagcttacagctgctgtgttcatcgctgctcgtccgttgtaccagaccctagctaatgctgcacagtcagctttcttgttcgcacataaccctgtacgagcagctagt			
>PR*unk*-10	9533-9832	44.7	10F: ttccctcagaaggtaag; 10R:tttacgtgctgcactaag†
ttccctcagaaggtaagtacggacgtaagtaatctattagcttacattcatgctactacagggttccagaaagctaacaaaggacgtaagcttgactcagcactagctaagcgtaccatcctaggggatgctagacgtttaacgttcactcagaaccgagctgaccagttcacgtaccaacagaacctatggagtgtacagctacagttcatgcagcacgtacataagatgttcttacagttagtagttgaccctacggtaagcgtagcatctttaggtaagcttagtgcagcacgtaaa			

>PR*unk*-11	9233-9532	46.7	11F: ggaactaacccttggg; 11R: tgctagattcaatgcc‡
ggaactaacccttggggtggctcgttcgctcaatcagctatgactctcggtactgttactgctatgttcggtcttgagtctaagttaggtactggtataagcactgacttagttactcagctgcaaga agcaggtgctactgaggacatgattgatataactatggatggtatcttcgggaaggttactgagcagatgtacggagaggaactggacttagcctctcgcttcagtccgataggtttcgtagga agtacactagggttgatgttcacacatgatggggcattgaatctagca			
>PR*unk*-12	8933-9232	45.7	12F: gggccgtctggttctttg; 12R: actcatgatatcatcaag‡
gggccgtctggttctttgtggaagactgctggtaacatggctagtatagctaaagcctttcattctaacgcagaggtatcggaggaagagggtcagttacttcttaatgaagcagctaacgtcttt gcagggttaaaggattatcagaggtacgagactgcacttaacttcggtgagtaccggactacagctggtcgtaagatagcagatatcagtacagagtccgctatacctttactgttcagtgtac ctcctaaggcagctcaacgttactacgatacccttgatgatatcatgagt			

* annealing temperature: 53°C, PCR buffer with 3mM Mg^{2+}
† annealing temperature: 55°C, PCR buffer with 3mM Mg^{2+}
‡ annealing temperature: 61°C, PCR buffer with 1.5 mM Mg^{2+}

Table S2: Q-PCR primer and amplicon sequences.

Primer Name	Primer Sequence (5’ to 3’)	Amplicon Sequence (5’ to 3’)
PSA-HP1_dF	TCTCTCGTCTTAATGACTTTCATCAT	GTATTTAATAAGTTCTTTAGTAAGTTCGTT
PSA-HP1_dR	TTCTTTCTCAACTTCCTGCTCTAA	

Table S3: Calculated and measured T_m for the polynucleotides forming the *unk* probe mix. The T_m was calculated for the composition of the hybridization-like buffer (HB) –1.718 M Na^+ and 35% formamide, and of the washing-like buffer II (WB) – 0.023 M Na^+.

		Measured T_m (°C) in HB	calculated T_m(°C)		%GC
			in HB	in WB	
unk1	probe	68.2			43.4
	target	73.9	73.8	70.4	
	hybrid	69.2			
unk2	probe	66.8			45
	target	71.2	74.4	70.9	
	hybrid	68.8			
unk3	probe	66.7			44.6
	target	70.9	74.3	70.8	
	hybrid	68.8			
unk4	probe	66.0			42.6
	target	70.2	73.4	70.0	
	hybrid	67.8			
unk5	probe	66.4			43
	target	70.5	73.6	70.1	
	hybrid	68.3			
unk6	probe	69.2			44.6
	target	72.2	74.3	70.8	
	hybrid	70.1			
unk-mix		Average probes = 67.2	Average = 74.0	Average = 70.5	
		Average targets = 71.5			
		Average hybrids =			
		Min probes = 66.0	Min =73.6	Min = 70.0	
		Min targets = 70.2			
		Max probes = 69.2	Max = 74.4	Max =70.8	
		Max targets = 73.9			
		ΔT_m probes = 3.2	ΔT_m = 0.8	ΔT_m = 0.8	
		ΔT_m targets = 3.7			
		Denaturation temperature = 85 °C			
		Hybridization temperature = 42 °C			
				Washing temperature = 42°C	

SI Figures

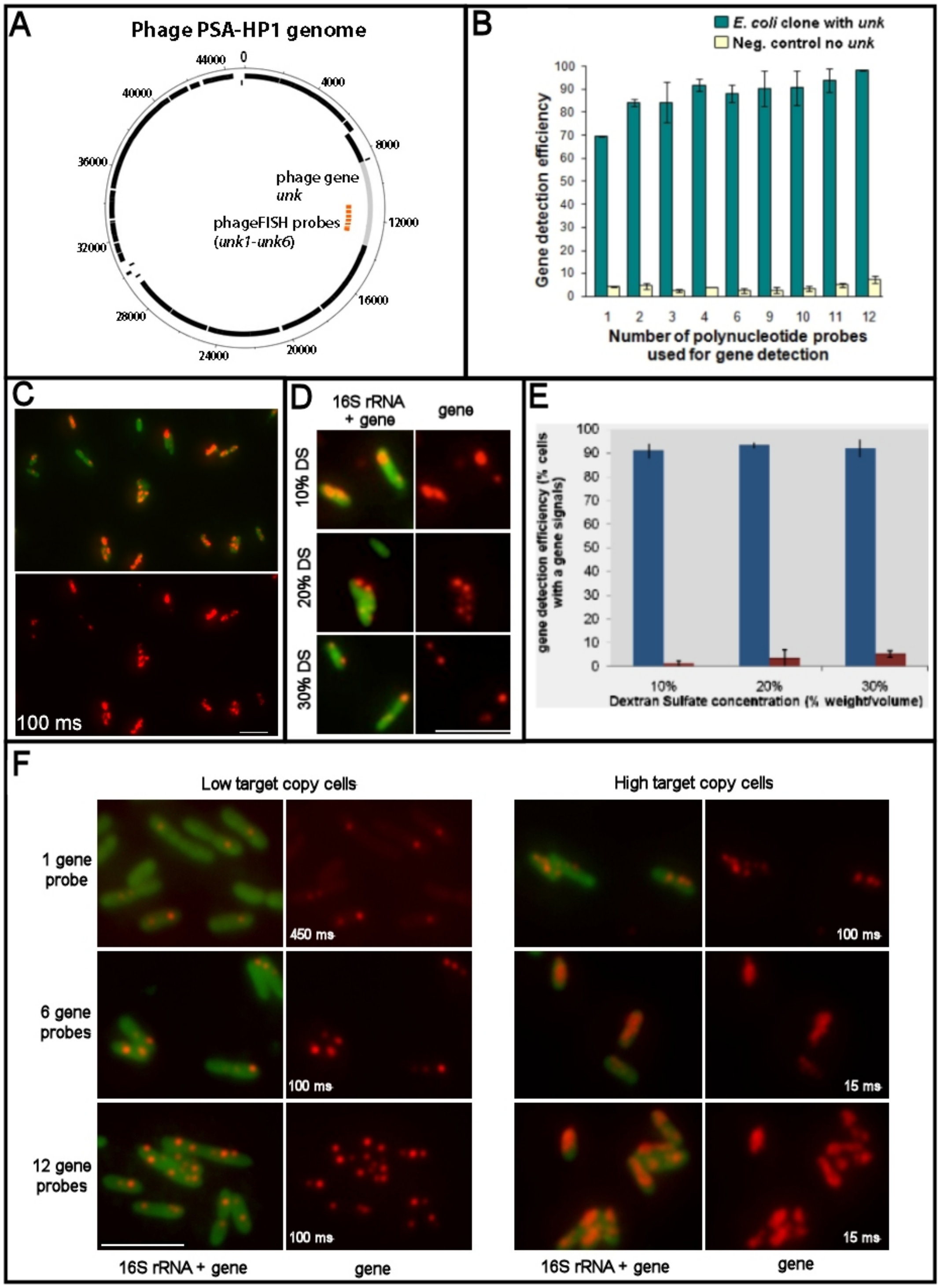

Figure S1: geneFISH protocol optimization. A: Genome map of phage PSA-HP1. The six (300 bp each) probe target regions are indicated in orange. Probes target unknown phage gene, *unk* (grey). B: Variation of the gene detection efficiency with increasing number of polynuclotide probes. *E. coli* low-target-copy clones (3–8 copies per cell) were hybridized with an increasing number of polynucleotide probes targeting the *unk* gene. As negative control (no *unk* gene), *E. coli* strain B/R cells were used. The detection efficiency is defined

as the fraction of all cells showing a gene positive signal. C: Detection of *unk* gene in high-target-copy cells using 3 polynucleotide probes – all the cells have a gene signal, resulting in 100% detection efficiency. Top image – overlay image between 16S rRNA signal and gene signal. Bottom image – gene signal. Scale bar = 5 µm. Exposure time [ms, milliseconds] is described for the gene image. D: Appearance of gene signals for different dextran sulfate concentrations (10%, 20% and 30%). All pictures were taken using the same exposure time. Concentrations of 20% and 30% dextran sulfate resulted in a much sharper signal as compared to 10%. Scale bar = 5 µm. E: Gene detection efficiency for different dextran sulfate concentrations (10%, 20%% and 30%). Blue bars = low target gene copy cells, red bars = negative control cells. While the detection efficiency was high for all concentrations, the background level (% of false positives in the negative control) increased with the dextran sulfate concentration. F: Variation of the gene (*unk*) signal intensity and spread through the cell with variation of the gene probe and target copy number. Scale bar = 5 µm. Exposure times [ms, milliseconds] are described for the gene images. The signal intensity increases with the increasing number of probes (higher exposure time was necessary when hybridizing with 1 probe). The signal spread and intensity increases with the increase in the target number, from dot-like for low target copy cells to whole cell signal for high target copy cells. For high target copy number cells, starting with ~6 probes, the signal does not increase anymore with the probe number, most likely due to a saturation of tyramide binding sites.

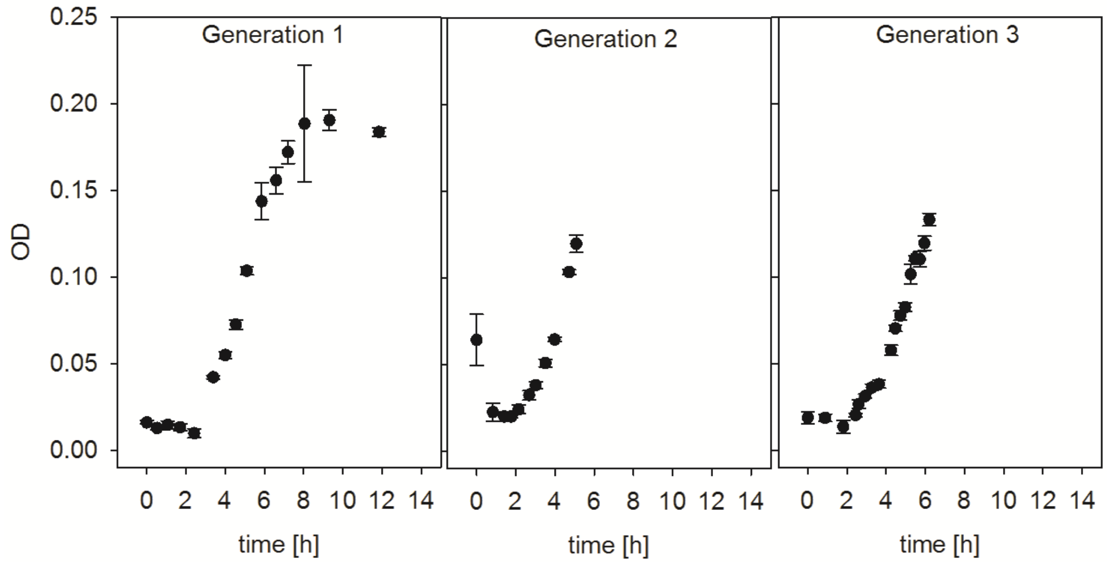

Figure S2: Pseudoalteromonas sp. H100 growth curves based on triplicate measurements. The bacterial host was physiologically acclimated for three generations resulting in 0.72 doublings per hour (± 0.06 doublings per hour, n = 3) during exponential growth. Error bars indicate standard deviation.

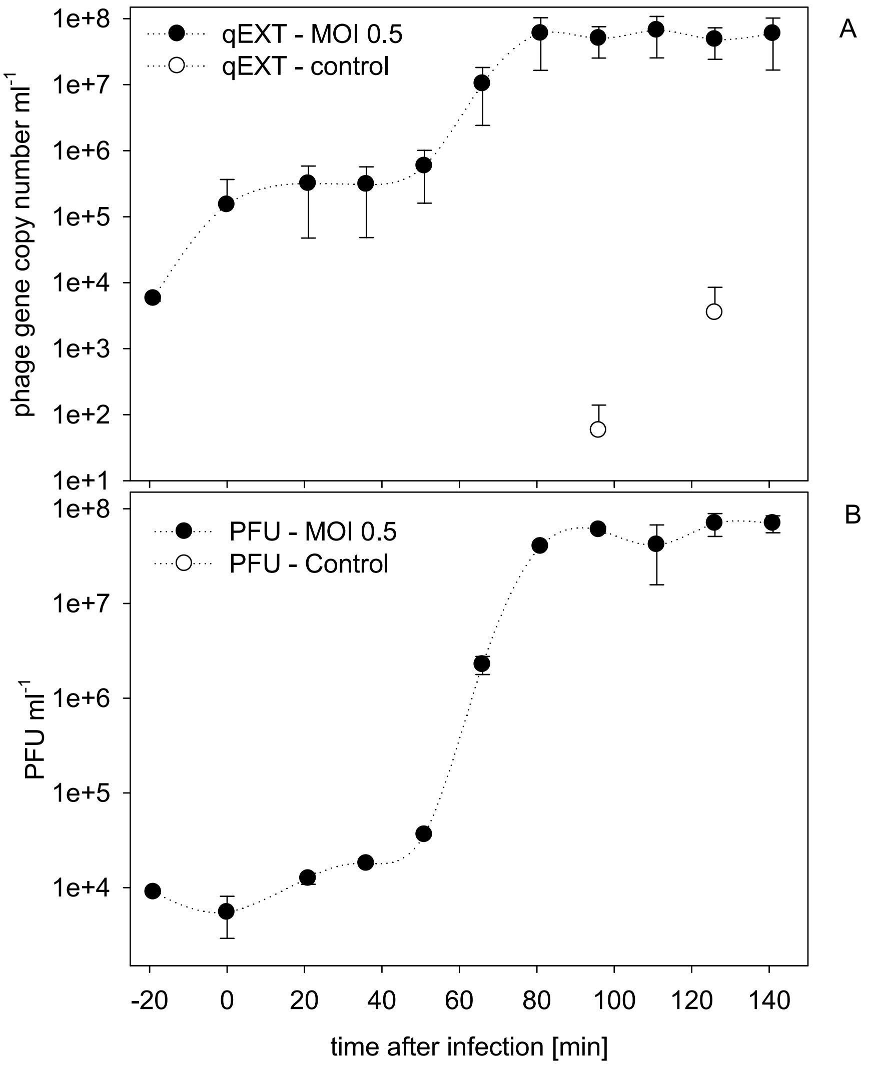

Figure S3: Virus assays including controls. A: Extracellular phage DNA as measured by quantitative PCR in infected (black circles) and control (white circles) cultures. B: Extracellular phage number as measured by plaque forming units (PFU) in infected (black circles) and control (white circles) cultures. Control data are zero unless plotted otherwise.

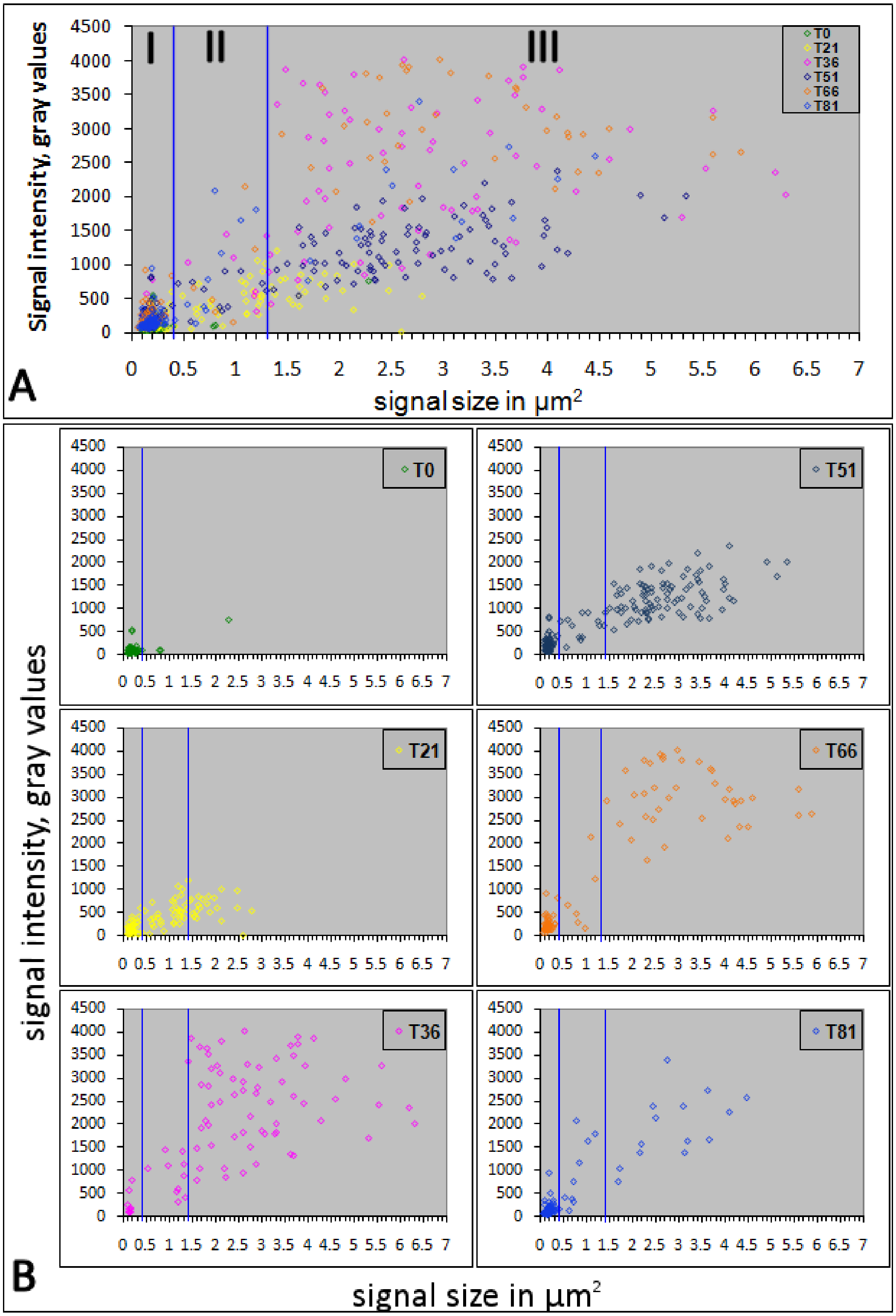

Figure S4: Determination of phage signal size classes and segregation of the two waves of infection. A. Plot of signal size versus signal intensity for T0 to T81. B. Plots of signal size versus signal intensity for each of the individual time points (from T0 to T81). Blue lines delimitate signal size classes. Class I (<0.4 µm^2): most probably new infections; Class

II (0.4 - 1.4 μm^2): most probably replicating infections; Class III (1.4 - 7.0 μm^2): most probably advanced infections. To establish the upper and lower limits of the smallest, first size class, we assumed that T0 signals represented new infections – these signals have both a small area and a low intensity (panel B). To establish the bounds of the largest, third size class, the first time point where both signal area and intensity were maximum (T36) was considered to represent advanced infections, i.e. late replication and assembly. All signals between those two size classes were considered as size class II, that is replicating infections – for examples, compare T0 with T21 and T36**.** While at T36 there were almost no class I signals, at T51 they reappeared and were abundant at T66 and T81. Furthermore, the class III signals decreased in abundance at T66 and T81. The re-appearance of class I signals in T51-T81 was assumed to represent new infection events by newly released mature phage particles and thus, a second wave of infection. All other T51-T81 signals were considered old infections from the first wave, in the process of phage maturation and release.

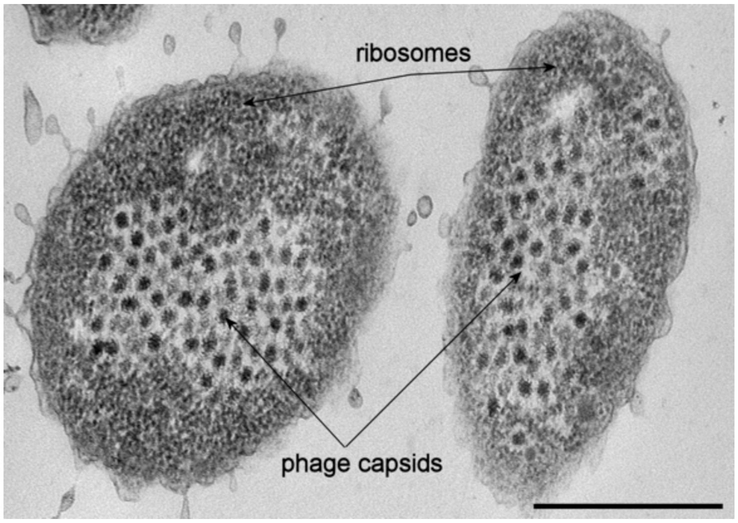

Figure S5: Localization of encapsidated phage and host cell ribosomes in TEM image of phage-infected Pseudoalteromonas cells from T66. Magnification 40,000x, scale bar = 500 nm.

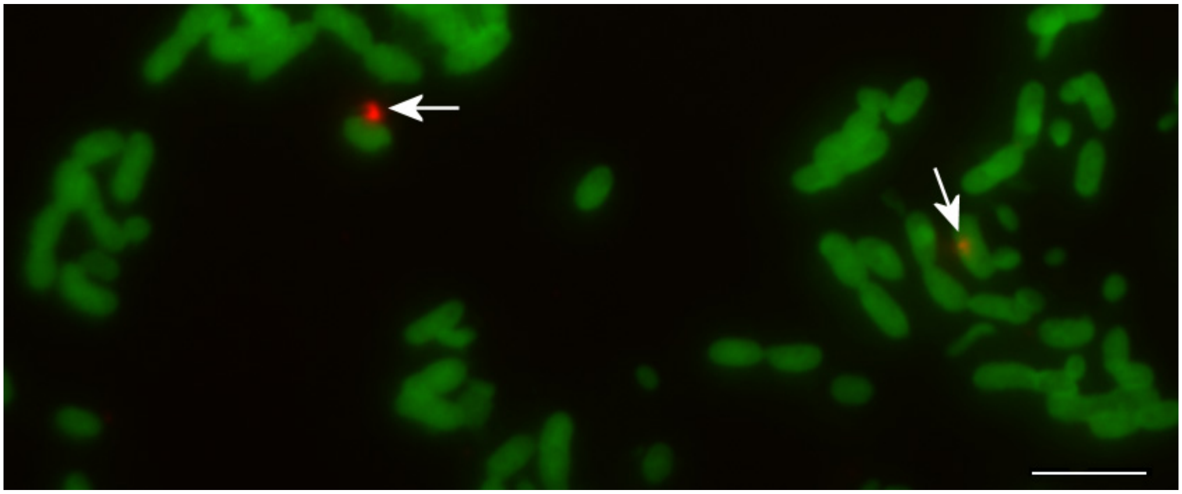

Figure S6: PhageFISH with the negative control gene probe (NonPoly350Pr) on infected cells from T81. The false positive events (white arrows) are all in the smallest signal size class and they amount to a background of ~2% from the cells. No false positives similar to the signals in the higher size class categories or to the cell bursts releasing phage particles are visible.

SI Appendix A

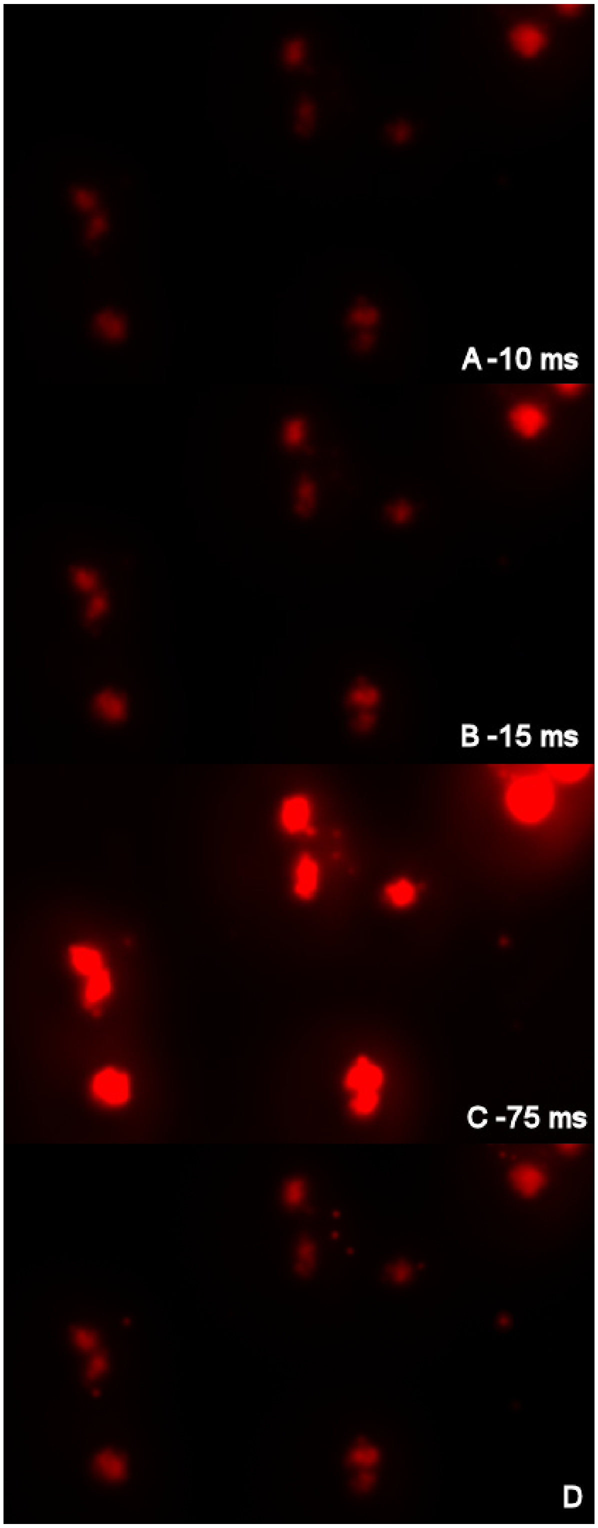

Fig. S7: Reconstruction of the $Alexa_{594}$ image T21 from Figure 2A by using the High Dynamic Range Imaging protocol. A–C – exposure time series; D – reconstructed image.

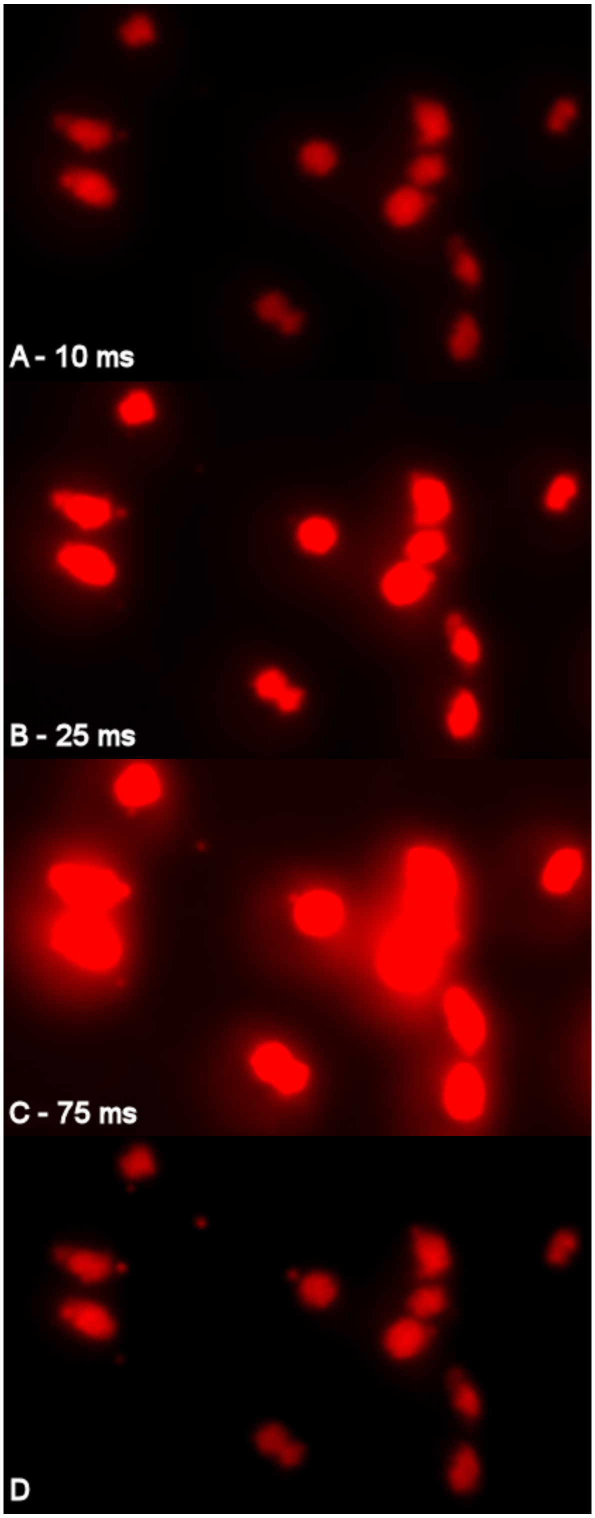

Fig. S8: Reconstruction of the $Alexa_{594}$ image T36 from Figure 2A by using the High Dynamic Range Imaging protocol. A–C – exposure time series; D – reconstructed image.

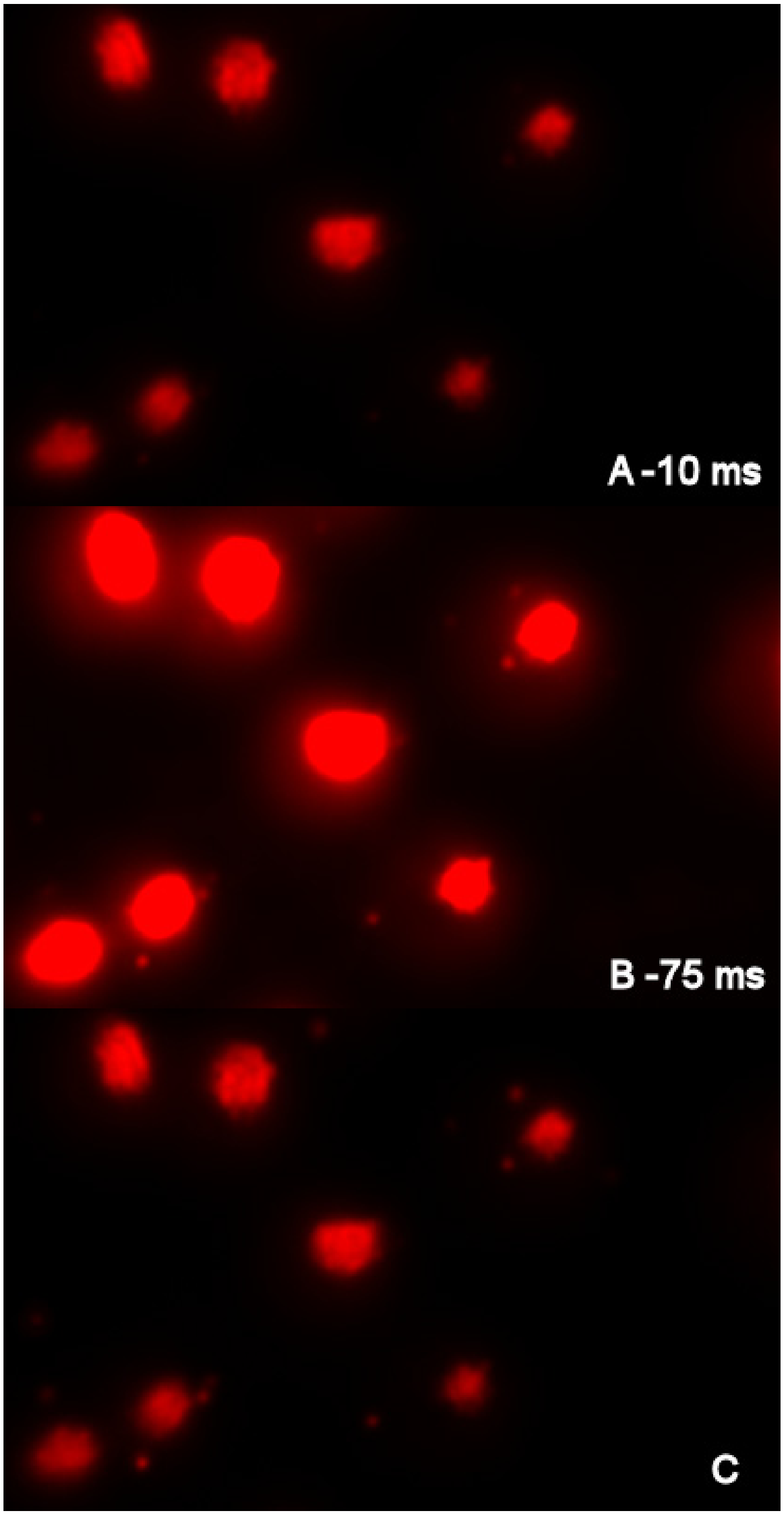

Fig. S9: Reconstruction of the $Alexa_{594}$ image T51 from Figure 2A by using the High Dynamic Range Imaging protocol. A–B – exposure time series; C – reconstructed image.

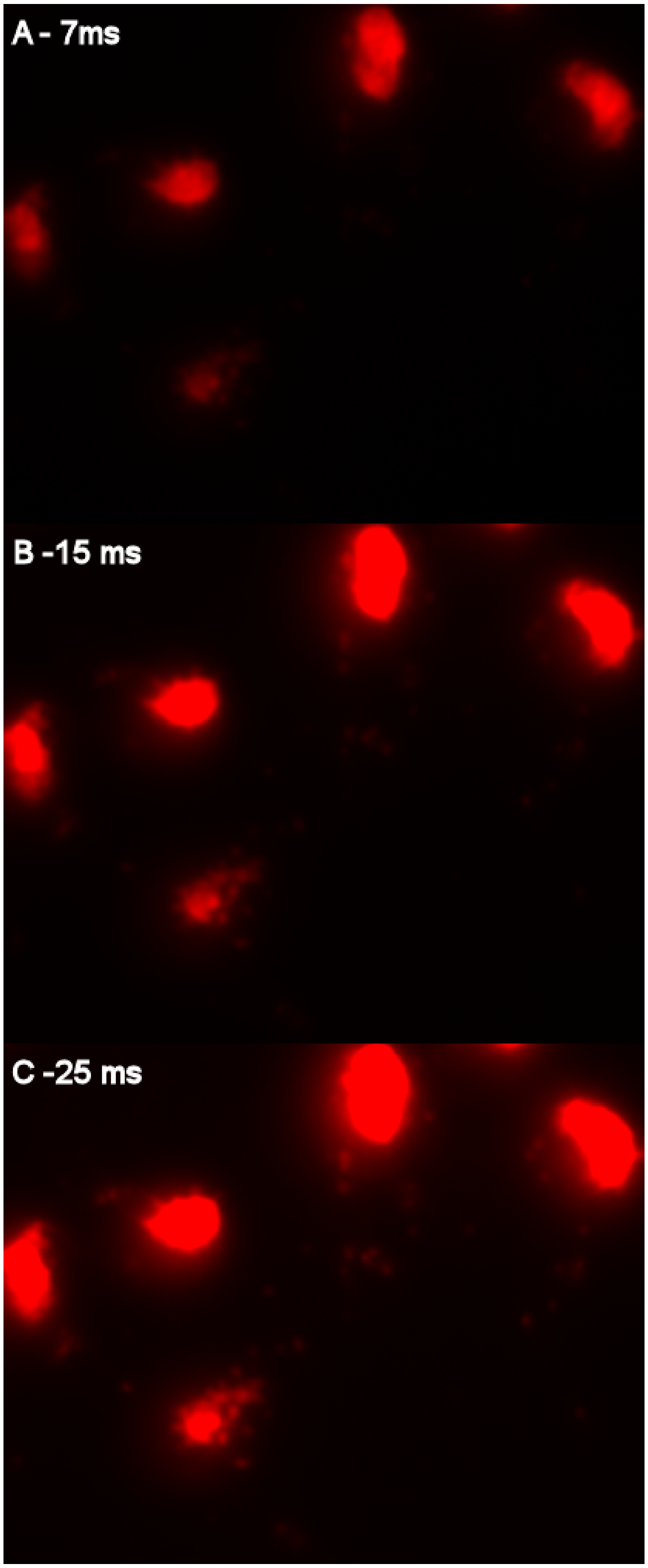

Fig. S10: Reconstruction of the $Alexa_{594}$ image T66 from Figure 2B by using the High Dynamic Range Imaging protocol. A–D – exposure time series; E – reconstructed image. Continued on next page.

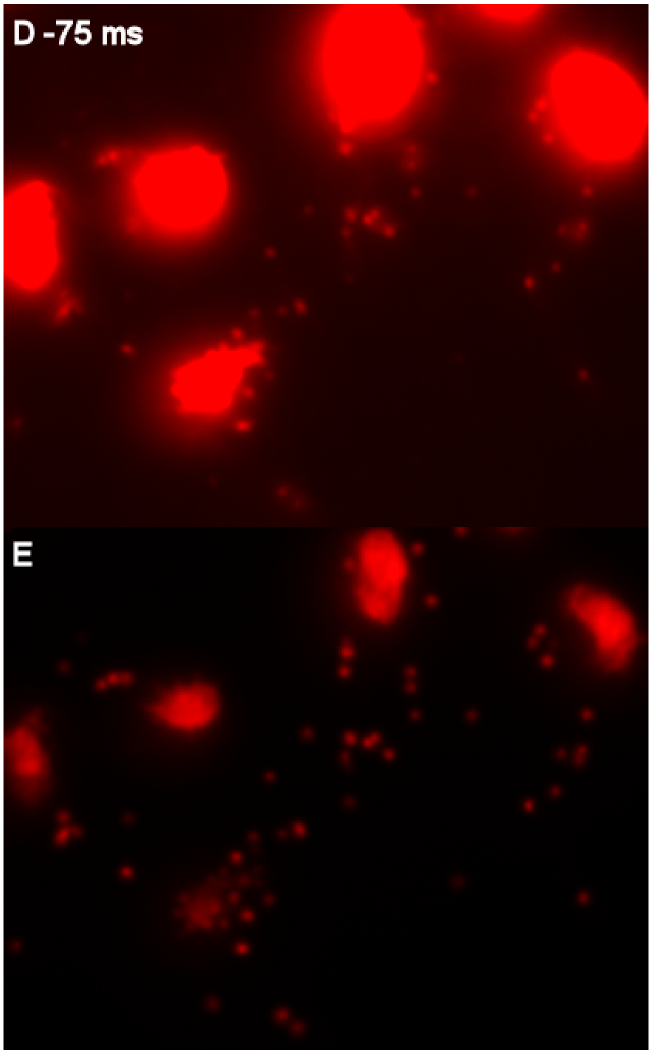

Fig. S10 (continued)

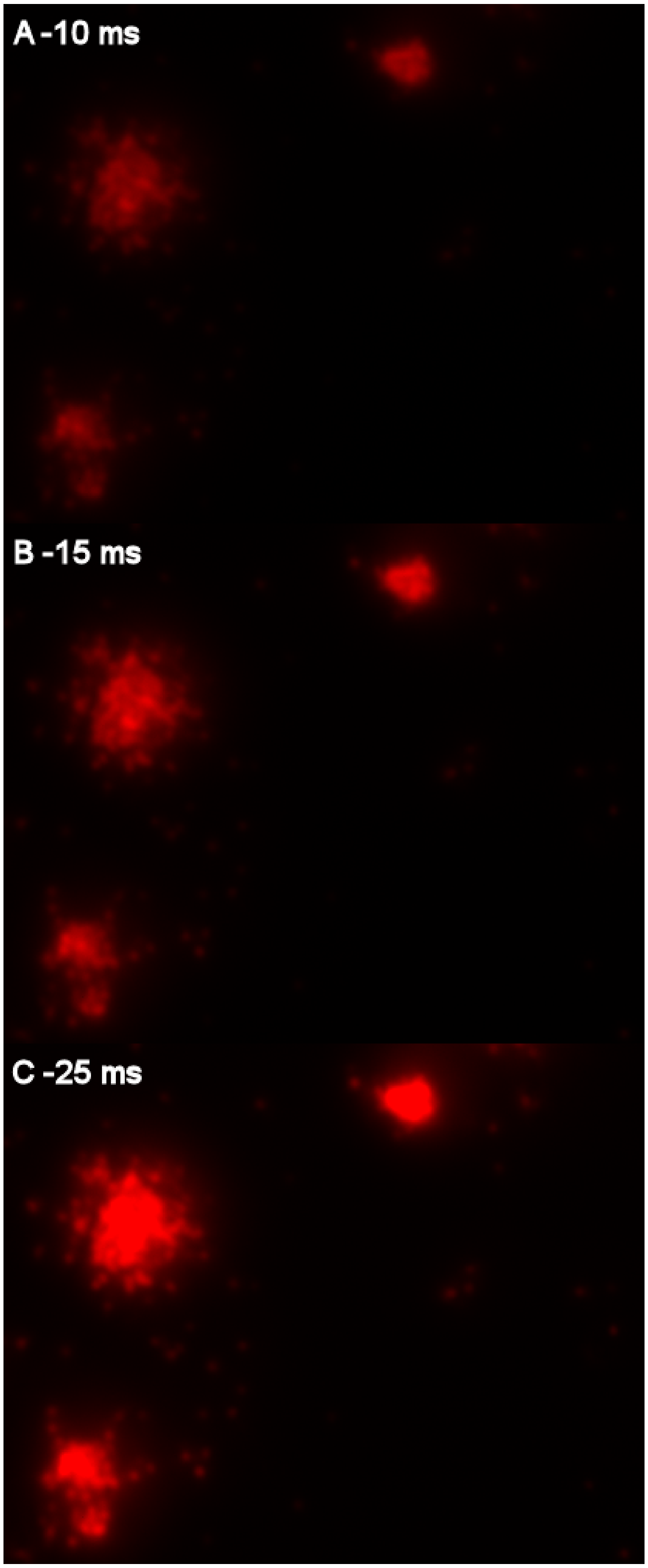

Fig. S11: Reconstruction of the $Alexa_{594}$ image T81 from Figure 2B by using the High Dynamic Range Imaging protocol. A–E – exposure time series; F – reconstructed image. Continued on next pages.

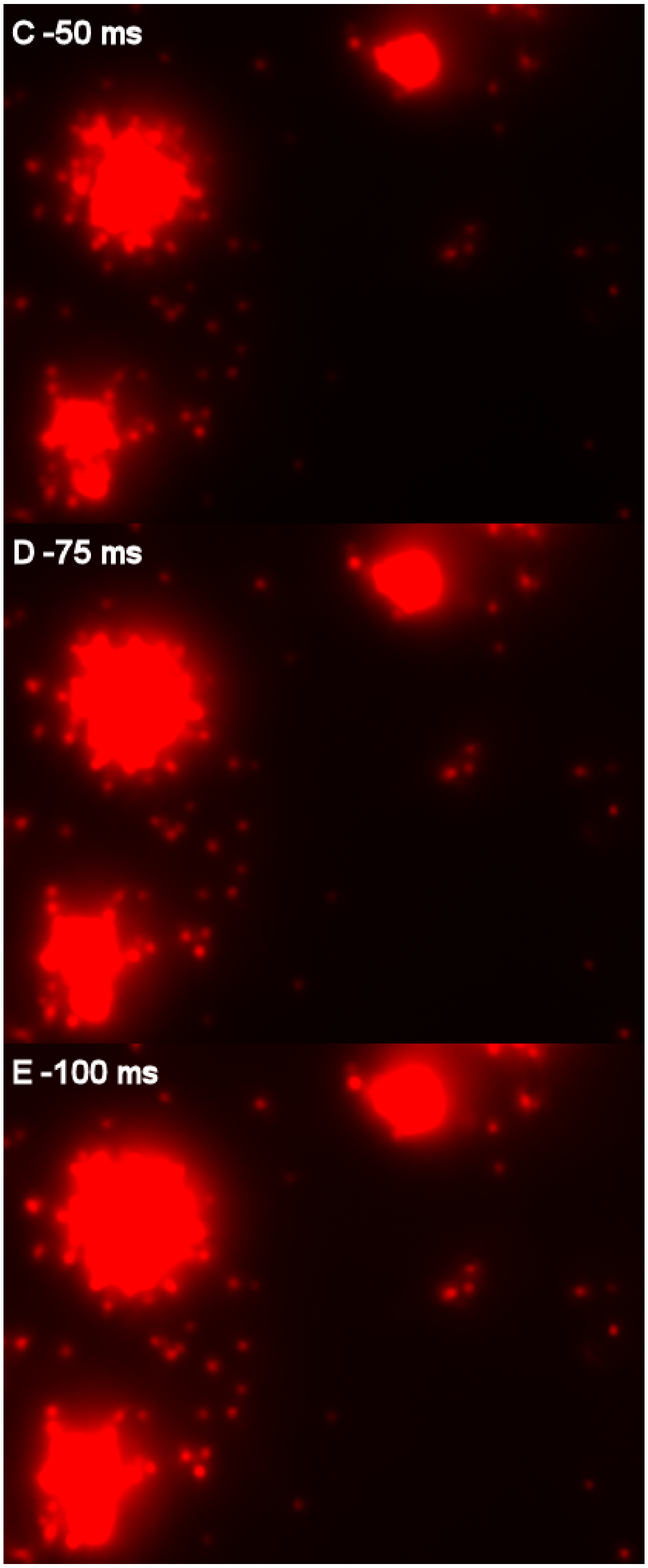

Fig. S11 (continued)

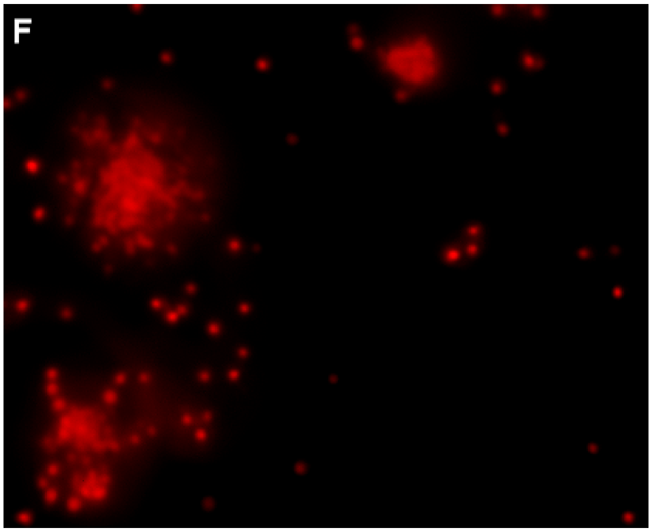

Fig. S11 (continued)

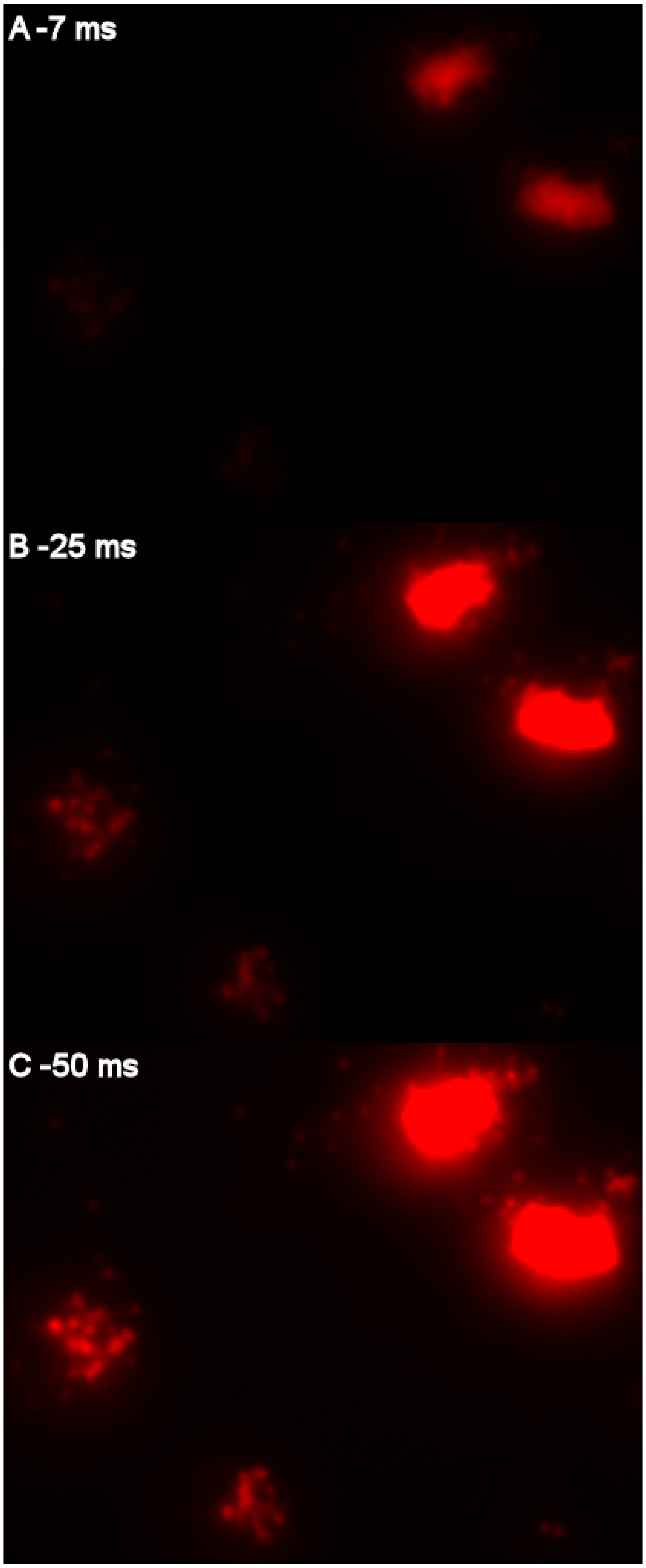

Fig. S12: Reconstruction of the $Alexa_{594}$ image T96 from Figure 2B by using the High Dynamic Range Imaging protocol. A–D – exposure time series; E – reconstructed image. Continued on next page.

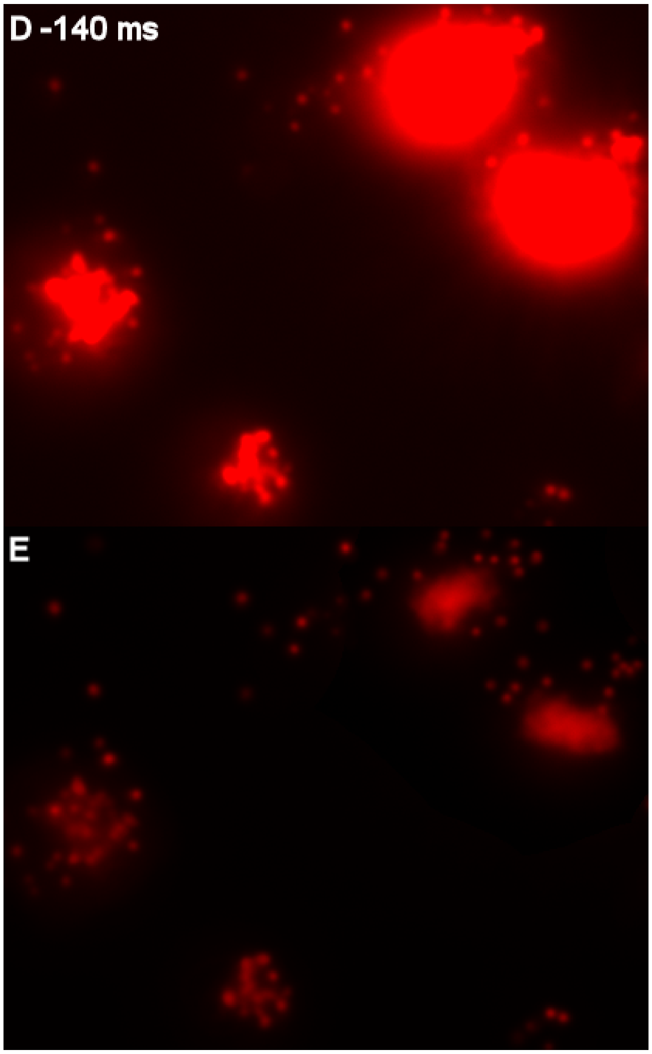

Fig. S12 (continued)

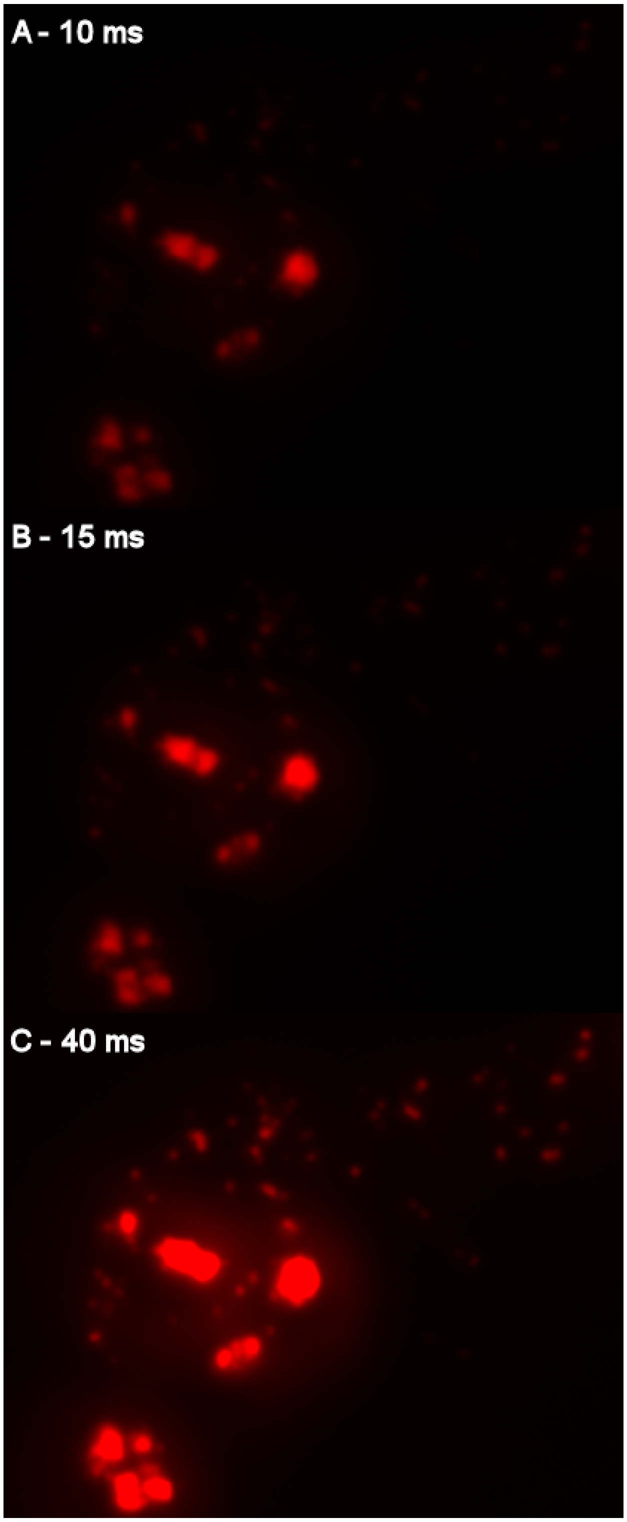

Fig. S13: Reconstruction of the $Alexa_{594}$ image T111 from Figure 2C by using the High Dynamic Range Imaging protocol. A–D – exposure time series; E – reconstructed image. Continued on next page.

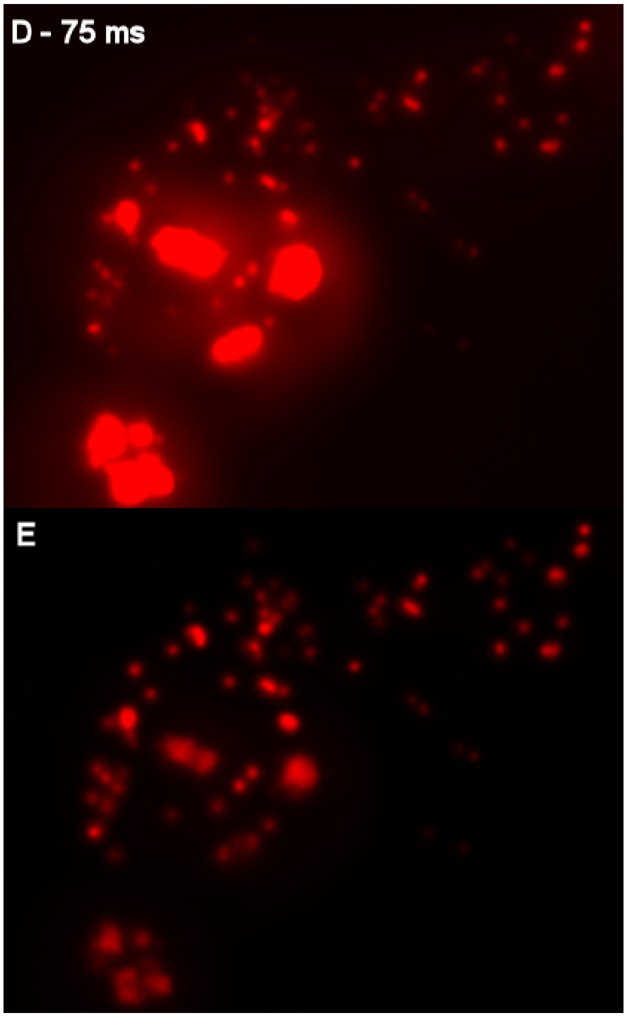

Fig. S13 (continued)

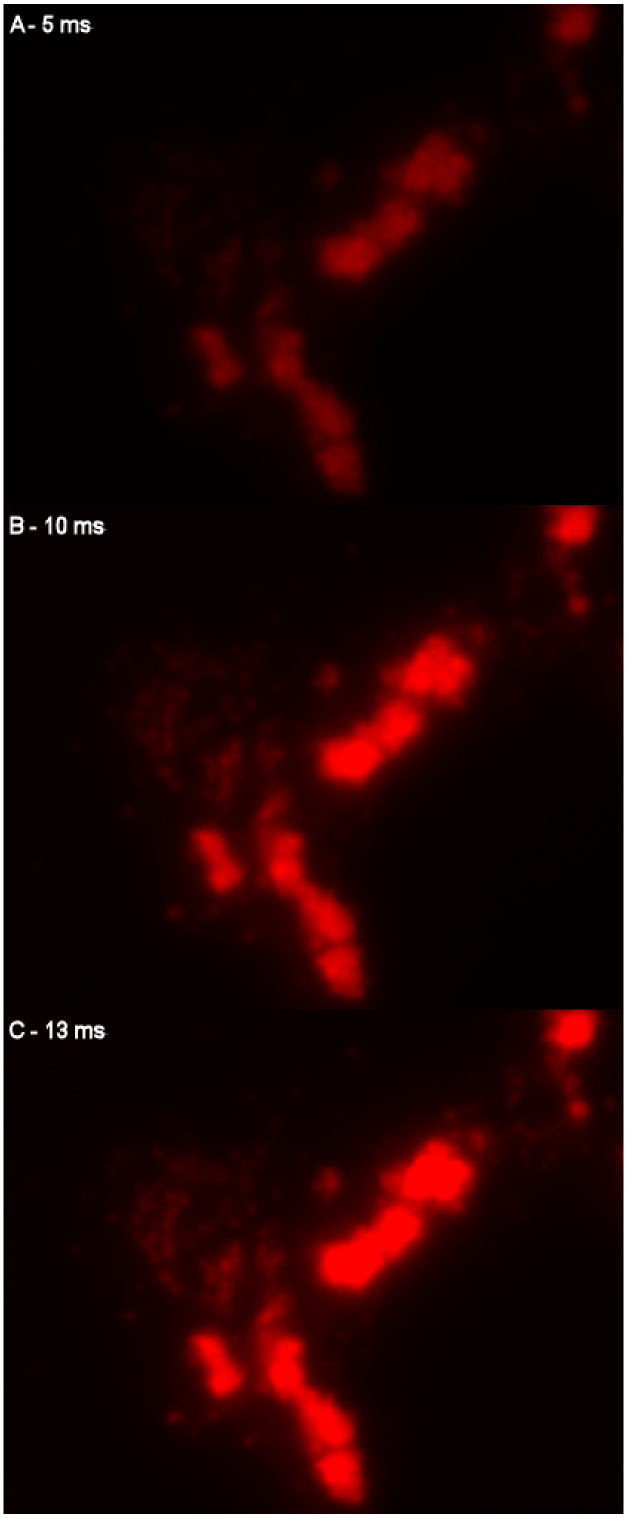

Fig. S14: Reconstruction of the Alexa$_{594}$ image T126 from Figure 2C by using the High Dynamic Range Imaging protocol. A–E – exposure time series; F – reconstructed image. Continued on next page.

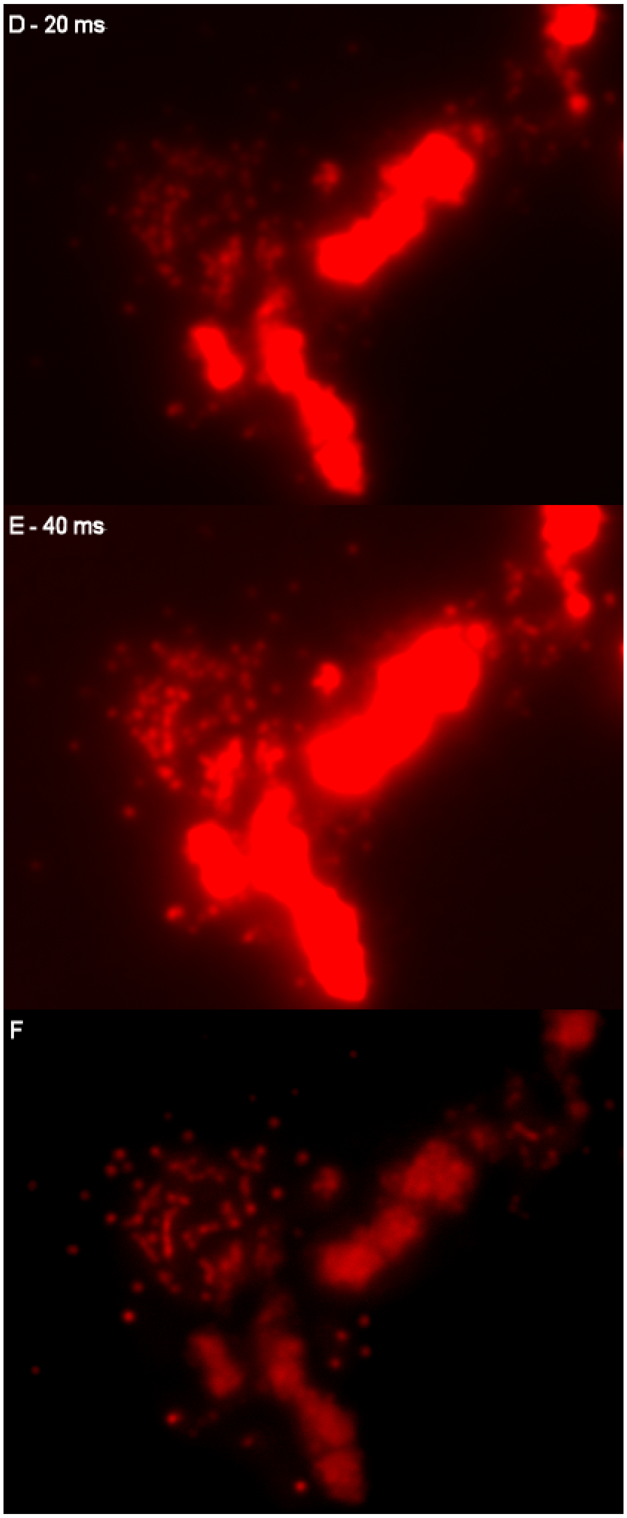

Fig. S14 (continued)

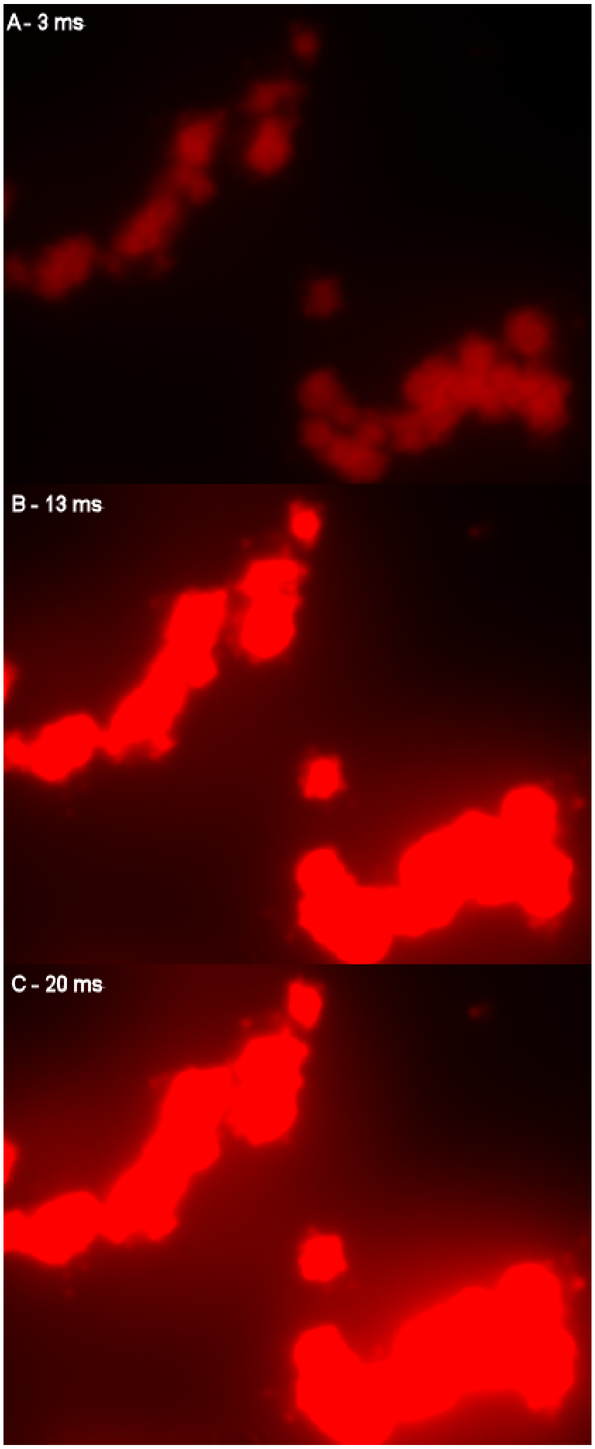

Fig. S15: Reconstruction of the $Alexa_{594}$ image T141 from Figure 2C by using the High Dynamic Range Imaging protocol. A–D – exposure time series; E – reconstructed image. Continued on next page.

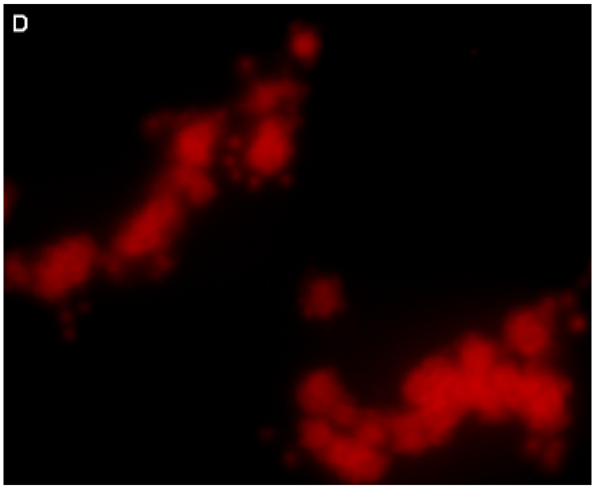

Fig. S15 (continued)
